# Neofunctionalization of zona pellucida proteins enhances freeze-prevention in the eggs of Antarctic notothenioids

**DOI:** 10.1038/ncomms12987

**Published:** 2016-10-04

**Authors:** Lixue Cao, Qiao Huang, Zhichao Wu, Dong-dong Cao, Zhanling Ma, Qianghua Xu, Peng Hu, Yanxia Fu, Yu Shen, Jiulin Chan, Cong-zhao Zhou, Wanying Zhai, Liangbiao Chen

**Affiliations:** 1 Key Laboratory of Exploration and Utilization of Aquatic Genetic Resources, Ministry of Education, College of Fisheries and Life Science, Shanghai Ocean University, Shanghai 201306, China; 2 Institute of Genetics and Developmental Biology, Chinese Academy of Sciences, Beijing 100101, China; 3 School of Life Sciences, University of Science and Technology of China, Hefei, Anhui 230026, China

## Abstract

The mechanisms by which the eggs of the Antarctic notothenioid fishes avoid freezing are not fully understood. Zona pellucida proteins (ZPs) are constituents of the chorion which forms a protective matrix surrounding the egg. Here we report occurrence of freezing temperature-related gene expansion and acquisition of unusual ice melting-promoting (IMP) activity in a family of Antarctic notothenioid ZPs (AnnotoZPs). Members of AnnotoZPs are shown to bind with ice and non-colligatively depress the melting point of a solution in a range of 0.26 to 0.65 °C at a moderate concentration. Eggs of zebrafishes expressing an AnnotoZP transgene show improved melting point depression and enhanced survival in freezing conditions. Mutational analyses in a representative AnnotoZP indicate the ZP domain and patches of acidic residues are essential structures for the IMP activity. AnnotoZPs, therefore, represent a group of macromolecules that prevent freezing by a unique ZP–ice interaction mechanism distinct from the known antifreeze proteins.

## Abstract (web summary)

Despite subzero temperature of their surroundings, eggs of Antarctic notothenioid fishes do not freeze. This study shows the expansion of genes related to freezing temperature in notothenioid which also acquired of ice melting-promoting zona pellucida proteins with specific structural properties.

## Graphical abstract

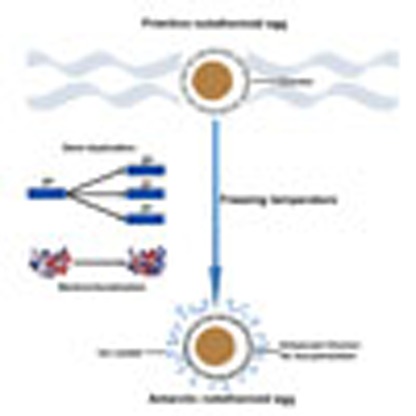

The marine habitats in Antarctica's near-shore waters regularly reach freezing temperatures (−1.9 °C) and are laden with environmental ice. These frigid waters are home to a highly endemic, cold-adapted ichthyofauna that is dominated by the perciform suborder Notothenioidei in both the number of species and biomass^1^. The five families comprising Antarctic notothenioids are descended from a temperate, benthic ancestor and have radiated throughout the Southern Ocean over the past 35 million years^1^,^2^.

The ecological success of Antarctic notothenioids in freezing seawaters is based on a series of cold-adaptive changes^3^. A striking example of such an adaptation is the acquisition of antifreeze glycoproteins (AFGPs)^4^,^5^, which depress the freezing point (FP) of the blood and body fluids through a non-colligative mechanism that is known as adsorption-inhibition^6^,^7^,^8^. In Antarctic notothenioids, AFGPs exist as a family of differently sized molecules at a combined concentration of 20 mg ml^−1^, which lower the FP of body fluids by ∼1.0 °C (ref. 9). The FP depression provided by AFGP6 at 30 mg ml^−1^ is 0.76 °C, whereas it is about 0.2 °C at 3 mg ml^−1^ (ref. 10). The acquisition of AFGPs, in combination with the ecological opportunities created by increased glacial and ice sheet activities, has enabled notothenioids to rapidly expand into icy habitats^11^.

In adult fishes, AFGPs are synthesized in exocrine pancreatic tissues and distributed throughout the body via circulation^12^. AFGP genes, however, are not expressed *in situ* in eggs, and it remains unknown whether other mechanisms exist that confer extra freeze prevention to reproductive cells. Nonetheless, studies of the spawned eggs of a few high-latitude Antarctic notothenioids suggest that the vitelline envelope (the chorion in fishes) provides a physical barrier to ice propagation^13^.

The chorion, a multi-lamellar, acellular, proteinaceous structure that surrounds the egg, is composed of a limited set of related glycoproteins called zona pellucida (ZP) proteins. These proteins share a conserved ZP domain that is ∼260 amino acids in length and contains eight highly conserved cysteine residues^14^,^15^. In mammals, ZP proteins have a role in egg-sperm recognition during fertilization, and thus constitute a reproductive barrier between species^16^,^17^. In fishes, the egg-sperm recognition function of ZP proteins is less regarded due to the presence of specialized micropyle for sperm to penetrate the chorion. Nonetheless, upon fertilization, the fish chorion is elevated and hardened, which deters the entry of multiple sperms^18^. The hardened chorion then forms a physical layer that protects the developing embryo.

In a previous study, transcripts of ZP genes from an Antarctic notothenioid, *Dissostichus mawsoni,* were sequenced and classified. The results revealed a multi-gene family encompassing all classes of vertebrate ZPs^19^. Seven of these ZP types exhibit substantial copy number amplifications in Antarctic notothenioids compared with temperate species, suggesting that Antarctic notothenioid ZP proteins (AnnotoZPs) may have acquired cold-adaptive functions. Regardless, it is not yet known whether the Antarctic-specific duplication of ZP genes is related to organismal survival.

We here provide experimental evidence indicating that AnnotoZPs possess unusual ice melting-promoting (IMP) activity. Evolution in freezing environments has led to substantial enhancement of this activity via alterations in the gene dosage. Such evolutionary changes likely provide extra freeze prevention to the eggs and presumably to other biological compartments when protection from the AFGPs is insufficient. The study also reveals a mechanism of ice-ZP interaction that non-colligatively lowers both the FP and the melting point (MP) of a solution.

## Results

### The ZP protein gene family in notothenioids

**Figure 1:**
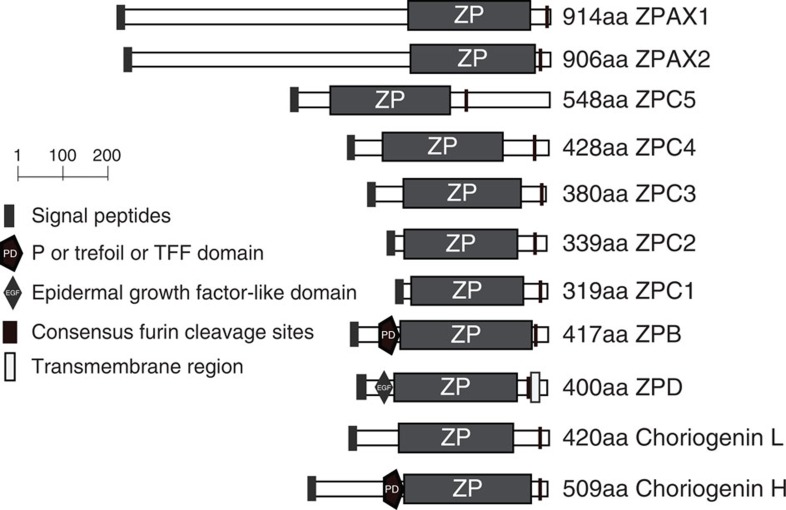
Schematic representation of the architecture of the ZP preproproteins of *D mawsoni*. Each protein represents a ZP type, which was named according to the results of a phylogenetic analysis of the vertebrate ZPs that were available in the public database. The structural domains are marked with different box patterns as indicated in the legend. The length of each bar is proportional to the size of the represented domain. The number of amino acids is shown to the right of each ZP type.

We retrieved 2,218 ESTs that contain a ZP domain from the published *D. mawsoni* ovary and liver EST databases^19^. When these ESTs were assembled, they yielded 105 ZP unigenes that collectively represent 11 ZP types, including ZPC1, ZPC2, ZPC3, ZPC4, ZPC5, ZPAX1, ZPAX2, ZPB, ZPD and choriogenins L and H. Full-length complementary DNAs (cDNAs) for each ZP type were obtained by sequencing the respective clones from the cDNA libraries, and the structural motifs encoded within these genes were identified using the SMART program^20^. Figure 1 shows the architecture of *D. mawsoni* ZP proteins (DmZPs). The DmZPs range in size from 914 aa (ZPAX1) to 319 aa (ZPC1), and they consistently contain an N-terminal signal peptide, a ZP domain, and a furin cleavage site, all of which are commonly present in vertebrate ZP proteins^21^.To determine whether the observed spectrum of DmZP types is common to other Antarctic notothenioids, we screened for homologous sequences in transcriptome data sets generated in a genome sequencing project undertaking in the laboratory. RNAs from multiple tissues from three Antarctic species, *Trematomus bernacchii*, *Gymnodraco acuticeps* and *Chionodraco hamatus*, were sequenced using Next-Generation sequencing technology^22^. We collected all transcripts annotated as ZP proteins. We identified all 11 ZP types in all three species and found many of them to be widely expressed among the tissues (Supplementary Fig. 1a). Transcripts representing the same set of AnnotoZP types were characterized from a basal notothenioid, *Eleginops maclovinus*, using reverse transcriptase–PCR. The results suggest that ZP types are highly conserved in notothenioids. The relevant ZP sequences and representative RNA sequencing data were deposited in public databases (Supplementary Table 1).

### Correlation between AnnotoZP expansion and freezing conditions

**Figure 2:**
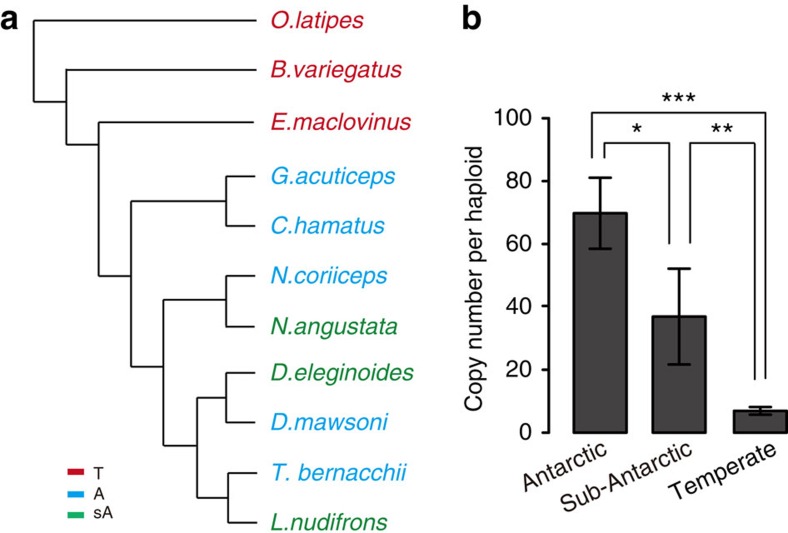
The levels of ZP gene copy number expansions in notothenioids were positively correlated with environmental freezing conditions. (**a**) A cladogram showing the phylogenetic relationships between the 11 species used in the ZP copy number study^55^. The group assignments for the species are denoted by A (Antarctic, blue), sA (sub-Antarctic, green) and T (temperate, red). (**b**) A one-way analysis of variance (ANOVA) test was performed to analyse ZP copy number variation among the Antarctic (*n*=5), sub-Antarctic (*n*=3) and temperate-water fish (*n*=3) groups. The copy numbers for each of the seven ZP types listed in Supplementary Table 2 were summed up for comparison. *Post hoc* multiple comparisons Tukey's honest significance tests were performed between the groups. Significant results are indicated by ‘***'(*P*=0.0012), ‘**'(*P*=0.00274) and ‘*'(*P*=0.0322).

To determine the relationship between the extent of ZP gene duplication and habitat temperature, patterns of ZP gene duplication were examined across 11 teleosts. The 11 species included an outgroup, *Oryzias latipes,* and ten notothenioid species, including two basal temperate species (*Bovichtus variegatus*, *E. maclovinus*), five Antarctic species (*D. mawsoni*, *Notothenia coriiceps*, *C. hamatus*, *G. acuticeps* and *T. bernacchii*) and three sub-Antarctic species (*N. angustata*, *Lepidonotothen nudifrons* and *D. eleginoides*), representing the three thermal evolutionary histories of notothenioid fishes (Fig. 2a). Using absolute quantitative real-time PCR, we determined the copy number of each ZP type in the haploid genome of each species (Supplementary Table 2). All ZP types were present at approximately single-copy levels in the temperate species, *O. latipes*, *B. variegatus* and *E. maclovinus*. However, the Antarctic and sub-Antarctic species had multiple copies of seven (ZPC1, ZPC2, ZPC3, ZPC5, ZPB, ZPAX1 and ZPAX2) of the 11 ZP genes. These seven ZP genes ranged from 3.5 to 15 copies in *D. mawsoni*, 3 to 25 copies in *N. coriiceps*, 3 to 20 copies in *C. hamatus*, 1 to 37 copies in *G. acuticeps*, 1 to 35 copies in *T. bernacchii*, 1.5 to 13 copies in *N. angustata*, 4 to 11 copies in *D. eleginoides* and 2 to 6 copies in *L. nudifrons*. When we compared the Antarctic and sub-Antarctic notothenioids, which currently occupy habitats south and north of the Antarctic Polar Front, respectively, we found that the northern (and thus warmer zone) species have lower copy numbers of these genes. One-way analysis of variance was performed to analyse differences in gene copy numbers in the three groups (Fig. 2b), and the results showed a positive correlation between ZP gene expansion and the severity of freezing temperatures.

We then examined the relationship between the extent of gene expansion and transcript abundance of the ZP types. In *D. mawsoni,* the 105 *Dm*ZP unigenes are unequally distributed among the 11 ZP types. The most prevalent type, DmZPC5 and which is also most extensively duplicated in *D. mawsoni* (Supplementary Table 2), contains 36 unigenes, and the second, DmZPAX1, contains 17, composing 35.2 and 18.6% of the total number (2,218) of ESTs, respectively. In contrast, the non-duplicated DmZPD and DmZPC4 genes, each of which is present with only one unigene, represent only ∼0.5% of the transcripts (Supplementary Fig. 1b). The correlation between ZP copy number and transcript abundance was further examined using real-time PCR in the ovary tissue from a different *D. mawsoni* specimen and found ZPC1 and ZPC5 are the two types producing the highest levels of transcripts (Supplementary Fig. 1c). With ∼9 copies, ZPC1 ranked the third in copy number in this species (Supplementary Table 2). In *T. bernacchii*, ZPC5, ZPC3 and ZPC2 are among the top duplicated ZP types, accordingly ZPC5 and ZPC2 are heavily transcribed and transcripts of ZPC3 are moderately abundant (Supplementary Fig. 1a). The data indicate that in general, the duplicated ZP copies are actively transcribed which result in high messenger RNA (mRNA) abundance of the ZP type.

### MP depression (MPD) by chorion proteins

**Figure 3:**
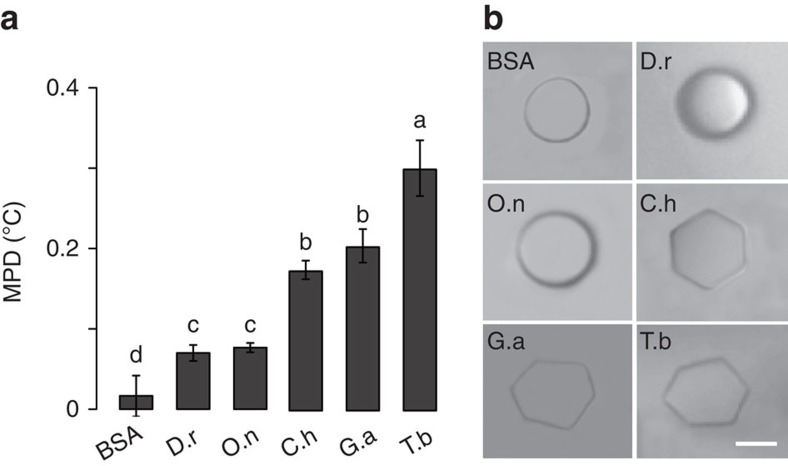
Melting point depression by chorion proteins of Antarctic notothenioids. (**a**) The melting point depression that was induced by bovine serum albumin (BSA) and the chorion extracts obtained from two tropical teleosts (*D. rerio* and *O. niloticus*) and three Antarctic notothenioids (*C. hamatus*, *G. acuticeps* and *T. bernacchii*). The data are shown as the mean±s.d. (*n*=3, biological replicates). Data denoted with different letters were significantly different from each other according to Tukey's multiple range test (*P*<0.0001). (**b**) The morphology of a single ice crystal was analysed at the freezing point of each of the protein preparations. All of the samples were measured at 3 mg ml^−1^ in TNE buffer. The MPD shown in **a** was obtained by subtracting the eqMP of TNE (−0.52 °C) from the MP that was detected using a Nanoliter Osmometer. Scale bar, 25 μm.

The Antarctic-specific expansion of ZP genes implies that AnnotoZPs might have cold-adaptive functions. One possibility is that these chorion proteins have acquired extra freezing-prevention activity, which is unnecessary for fishes inhabiting warmer climates. To examine this possibility, we isolated chorions from the oocytes of three Antarctic notothenioids (*T. bernacchii*, *G. acuticeps* and *C. hamatus*) and two tropical fishes (*Danio rerio* and *Oreochromis niloticus*) and extracted the chorion proteins from them (Supplementary Fig. 2a,b). The chorion proteins were then dissolved in TNE (50 mM Tris-HC1, 125 mM NaC1, 10 mM EDTA, pH 7.2) and subjected to nanoliter osmometer analysis to determine the FP, MP, and patterns of ice crystal growth associated with the proteins. The results are shown in Fig. 3. At a concentration of 3 mg ml^−1^, the ice in the solutions containing the Antarctic notothenioids chorion proteins melted earlier and thus at a lower temperature than the ice in the solutions containing chorion extracts from the tropical fishes. These results demonstrated that the chorion proteins caused MPD; we refer to this activity of ZP proteins as IMP activity. MPD is defined as the difference between the observed MP of a ZP solution and the equilibrium (eq) MP of the solvent, which was −0.52 °C for TNE and −0.68 °C for 1 × TBS (used in later experiment), respectively. The extent of MPD varied according to species, and the MPDs of the Antarctic species were generally higher than the MPDs of other species (Fig. 3a). The observed FP was very close to the MP in each chorion sample, indicating that FP and MP were simultaneously depressed. The ice crystals grown in the Antarctic notothenioid chorion solutions adopted a hexagonal shape. In contrast, the crystals in the bovine serum albumin (BSA) solutions or the chorion extracts obtained from tropical fishes were mostly round in shape (Fig. 3b), indicating that the proteins in the Antarctic samples possessed stronger ice-faceting activity. Occasionally, when higher protein concentrations (that is, 4 mg ml^−1^) were used in the tests, ice-faceting (that is, hexagonal edges) were observed in zebrafish chorion extracts, indicating that a primordial ice-interaction activity was associated with teleost chorion proteins. The unusually lowered MPs and the stronger ice-faceting activities that we observed in the Antarctic notothenioid chorions prompted us to explore the functions of individual AnnotoZPs in ice melting.

### Non-colligative MPD by recombinant AnnotoZPs

To determine the role that individual AnnotoZPs have in MPD activity in chorion extracts, we expressed five recombinant *D. mawsoni* ZP genes representing the four major types (ZPAX, ZPB, ZPC, ZPD) of ZP proteins in the Chinese Hamster Ovary (CHO) cell line and then purified them from whole cell lysates using antibody-based affinity chromatography (Supplementary Fig. 2c–e). The purified proteins were dissolved in 1 × TBS (50 mM Tris-HCl, 150 mM NaCl, pH 7.4), and their ion levels were examined using ion chromatography to ensure the absence of unwanted osmolyte contaminations. We then analysed the proteins to determine their MPD and ice-faceting activity. Substantial MPD levels were detected for all five recombinant DmZPs (Table 1), which showed values ranging from 0.26±0.02 °C (ZPC1) to 0.65±0.17 °C (ZPAX1), all of which were significantly higher than the levels for the same concentration (3 mg ml^−1^ w/v) of BSA (0.007±0.02 °C). The observed MPD for each of the recombinant ZP proteins far exceeded the colligative MPD value, which can be approximately estimated from the observed MP of BSA in this test (Table 1). The MPD caused by the ZP proteins appeared to be concentration-dependent within the range of tested concentrations (1 to 4 mg ml^−1^), as shown for DmZPC5 in Supplementary Fig. 3a,b. This range is similar to the thermal hysteresis caused by high concentrations of AFGPs.

To exclude the possibility that the MPDs observed for the AnnotoZPs resulted from osmolyte contamination, we used an alkaline solution to denature the proteins before the MPD assays. We expected to observe diminished MPDs after the alkaline treatment was applied if the MPD activity was a *bona fide* function of the ZP protein. Indeed, in each AnnotoZP we tested, the MPD activity was dramatically reduced to background levels. As expected, the BSA solution, which did not show any IMP activity, displayed near identical MPD values when tested with or without alkaline treatment (Supplementary Fig. 3c).

ZP proteins are naturally secreted proteins. To reveal whether the secreted ZP proteins possess MPD activity, we isolated the secreted DmZPC5 and DmZPAX1 from the culture media of the ZP-expressing CHO cells (Supplementary Fig. 4a). The molecular weight of the secreted DmZPC5 was about 100 kD, significantly larger than the non-secreted form for an unknown mechanism. MPD activities were detected to be 0.26 °C and 0.29 °C for the secreted DmZPC5 and DmZPAX1, respectively, at 3 mg ml^−1^, and the activities diminished after alkaline treatment (Supplementary Fig. 4b). The MPD activity of secreted DmZPC5 was 0.38 °C at 5 mg ml^−1^. The results indicated that naturally secreted ZP proteins are active in MPD, and their FP/MPD activities are in a similar range as that of thermal hysteresis activity of AFGPs when measured at similar weight/volume concentrations.

One puzzling phenomenon related to the thermal behaviour of the AnnotoZP proteins was thermal hysteresis, which is defined as the temperature difference between the MP and FP of an antifreeze protein (AFP) solution and is a hallmark of all known AFPs^23^, was barely detectable for all AnnotoZPs (Table 1). This evidence suggests that a different mechanism underlies the non-colligative MP/FP depression that is induced by AnnotoZPs than is involved in adsorption-inhibition mechanism. Nevertheless, when ice crystals were grown in solutions containing recombinant AnnotoZPs, they were all hexagonally shaped, as observed in the whole-chorion extracts of the Antarctic fishes, suggesting that each recombinant AnnotoZP possesses the ability to interact with ice. AnnotoZPs are novel ice binding proteins that are capable of promoting ice melting at temperatures lower than the colligative MP of a solution.

### AnnotoZP transgene confers IMP function to zebrafish egg

**Figure 4:**
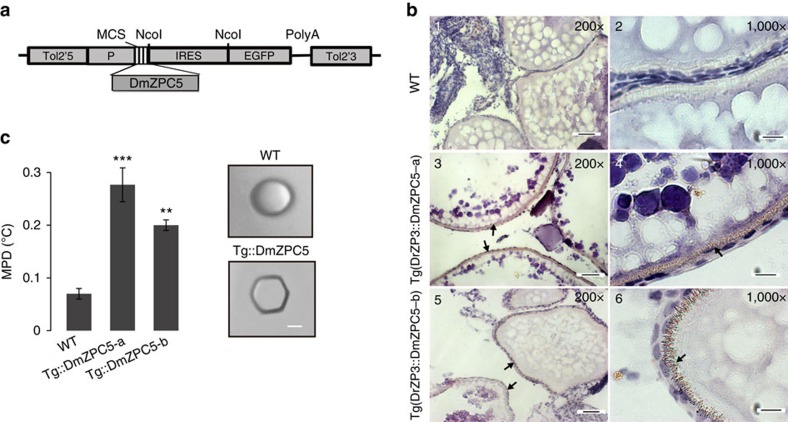
DmZPC5 transgenic zebrafish showed improved melting point depression compared with that observed in the chorions of the tropical fish. (**a**) The construct used to establish the transgenic line. The letter ‘p' represents the DrZP3 promoter. (**b**) Immunohistochemical staining showing the localization (the sheets of brown dots indicated by the arrows) of the DmZPC5 transgene product in the chorion of the two transgenic fishes (DrZP3::DmZPC5-a (pictures 3, 4), -b (pictures 5, 6)) and the lack of staining in wild-type (WT, pictures 1, 2) chorions. Scale bar for pictures 1, 3, 5 is 40 μm and scale bar for pictures 2, 4, 6 is 10 μm. The tissues shown were taken from adult fish ovary. (**c**) Enhanced MPD (left panel) and ice-faceting activity (right panel) were detected in the transgenic zebrafish chorions. The bars represent the mean±s.d. (*n*=3). Scale bar, 25 μm. Statistical analysis were performed with unpaired Student's *t*-test and significant results are indicated by ‘**'(*P*=0.0004) and ‘***'(*P*=0.0001). A same concentration (3 mg ml^−1^) of total chorion extract from each sample was used for the MPD assay.

Transgenic studies were performed to examine whether the highly active AnnotoZPs could confer IMP activity to the egg chorions of other fishes. Two independent female founders were generated in which DmZPC5 and green fluorescence protein (EGFP) were polycistronically expressed under the zebrafish ZP3 promoter (Fig. 4a). We performed an immunohistochemical assay using a rabbit polyclonal antibody that was specifically raised against DmZPC5. The results showed that the transgene was expressed in oocytes and correctly distributed to the chorion and cortex granules, following pattern similar to that of the endogenous ZP products in zebrafish^24^ (Fig. 4b). Total chorion proteins were extracted from the oocytes of transgenic animals and assayed to determine MPD using a same concentration (3 mg ml^−1^) of the chorion proteins for each sample. The chorions of both transgenic fish lines resulted in significantly lower MPs and sharper faceted ice edges than the proteins from the wild-type zebrafish (Fig. 4c). These results confirmed that individual AnnotoZPs conferred MPD to the chorion of another fish species.

**Figure 5:**
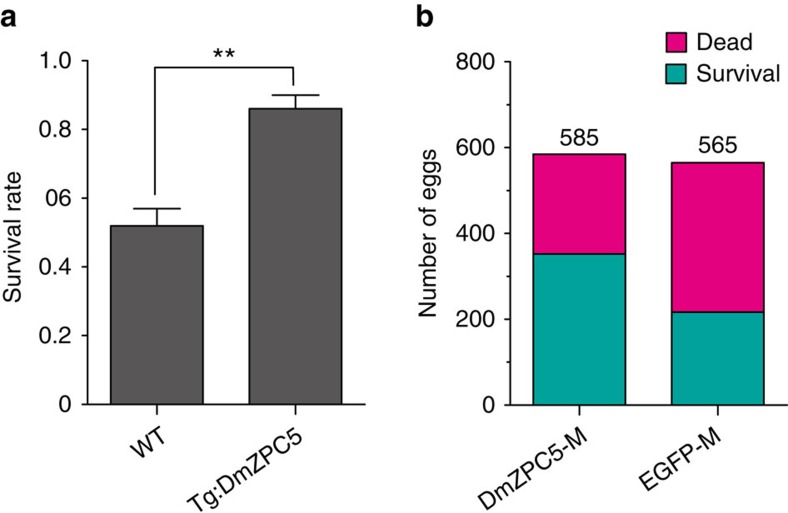
Presence of DmZPC5 *in vivo* or *in vitro* improves zebrafish egg survival in freezing conditions. (**a**) The survival rates of the transgenic (Tg:DmZPC5) and wild-type (WT) zebrafish eggs when exposed to −2 °C for 20 min. Statistical significance was analysed by unpaired Student's *t*-test (‘**'indicates *P*=0.0039). (**b**) The number of developing eggs that survived or died after exposed to 0 °C for 40 min in the presence (DmZPC5-M, ∼5 mg ml^−1^) or absence (vector-M) of DmZPC5. Serum-free culture media of the CHO cells that express DmZPC5 or harbour an empty vector were collected, concentrated and dialysed against the E3 hatch medium. Two-millilitres of the dialysed media were added to replace the original hatch media. The eggs were treated at 0 °C for 40 min. The assays were repeated three times with different batches of protein preparations and fertilized eggs.

To check whether presence of DmZPC5 would benefit egg survival in freezing conditions, we exposed fertilized eggs of the transgenic and the wild-type zebrafishes to a freezing temperature (−2 °C) for a duration of 20 min. Significantly higher survival rates were observed in the transgenic fishes than in the wild-type fishes (82.5% and 52.1% respectively) (Fig. 5a). Similarly, when the recombinantly expressed, secreted DmZPC5 (∼5 mg ml^−1^) was present in the hatch medium, greater number of fertilized eggs survived a mild freezing condition (0 °C for 40 min) than the eggs hatching in the same medium without DmZPC5 (Fig. 5b).

### The structural bases of IMP activity of the AnnotoZPs

**Figure 6:**
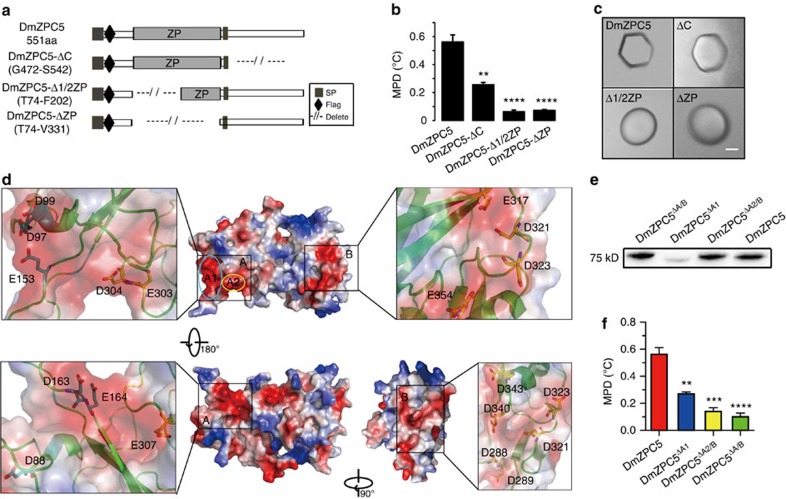
Structure–function analyses to determine the structural basis for the ice melting-promoting activity of the AnnotoZPs. (**a**) Schematic presentation of the structural domains of the native DmZPC5 (top) and three serial deletions (the lower three). The region of amino acids that are deleted was shown under the name of each mutant. (**b**) Melting point depression was analysed in the native and mutant DmZPC5. The bars represent the mean±s.d. (*n*=3, biological replicates). Statistical analyses were performed with the unpaired Student's *t*-test. Significant results are indicated by ‘****'(*P*=0.0001) and ‘**'(*P*=0.0002). (**c**) Alterations in the morphology of single ice crystals that were grown in solutions of DmZPC5 mutants. Scale bar, 25 μm. (**d**) The predicted electrostatic potential on the surface of DmZPC5. Red represents negatively charged patches, and blue represents positively charged patches. (**e**) Purified mutant proteins and native DmZPC5 variants analysed by western blot on reducing SDS–PAGE gels. (**f**) Melting point depression was measured in the site-mutated variants of DmZPC5 and compared with the results for native DmZPC5 at 3 mg ml^−1^ for each sample. The bars represent the mean±s.d. (*n*=3, biological replicates). Statistical analyses were performed with the unpaired Student's *t*-test. Significant results are indicated by ‘****'(*P*=0.0003), ‘***'(*P*=0.0004) and ‘**'(*P*=0.0015).

To analyse the structural bases of the ice-faceting and IMP activities associated with the AnnotoZPs, we performed functional studies in which we deleted the domains of one AnnotoZP type, DmZPC5 (Fig. 6a and Supplementary Fig. 2d,e for the protein products). When the region that lies C terminal to the ZP domain was deleted (DmZPC5-ΔC), MPD activity was decreased by ∼50%, but the ice-faceting activity persisted. In contrast, when we either completely (DmZPC5-ΔZP) or partially deleted (DmZPC5-1/2ΔZP) the ZP domain, the MPD was reduced by 90%, to the same level as that of the vector control, and ice-faceting activity was completely abolished (Fig. 6b,c).

Structural modelling of DmZPC5 indicated that the surface of DmZPC5 contained two acidic patches, referred to as acidic patch A and B, located on two adjacent sides of the molecule (Fig. 6d, Supplementary Fig. 5). Patch A was localized to the N terminus of the ZP domain and was divided into two sub-areas, A1 and A2, according to its distance from the N terminus. A1 contains six acidic residues, D88, D97, D99, E153, D163 and D164, whereas A2 possesses three acidic residues, E303, D304 and E307 (Fig. 6d). Patch-B is located on the C-terminal side of the ZP domain and consists of eight acidic residues: D288, D289, E317, D321, D323, D340, D343 and D354 (Fig. 6d). Similar electrostatic surface potentials were identified on the surfaces of DmZPAX1 and EmZPC5, but much weaker on ZFZP3C, an MPD-ineffective ZP in zebrafish (Supplementary Fig. 6a), suggesting that the acidic surface patches may have roles in MPD. We generated three variants containing multi-residue mutations from native DmZPC5 by replacing the acidic residues with neutral residues. In DmZPC5^ΔA1^, six residues were replaced, including D88N, D97N, D99N, E153Q, D163N and E164Q. In DmZPC5^ΔA2/B^, 11 residues were substituted, including D288N, D289N, E303Q, D304N, E307Q, E317N, D321N, D323N, D340N, D343N and E354Q. Finally, in DmZPC5^ΔA/B^, amino acid substitutions were performed at all of the acidic residues (Supplementary Fig. 5). Structural modelling of the DmZPC5 mutants indicated that the mutations abolished the acidic patches (Supplementary Fig. 6b). These three mutant proteins, and the native DmZPC5 were expressed in CHO cells and purified to homogeneity (Fig. 6e, Supplementary Fig. 7), and assayed to determine their effect on MPD at a same concentration (3 mg ml^−1^). We found that the MPD induced by DmZPC5^Δ^ was substantially reduced and that the MPD induced by DmZPC5^ΔA2/B^ and DmZPC5^ΔA/B^ was nearly completely lost (Fig. 6f). In the latter two mutants, the MPD levels were similar to those observed for the DmZPC5 mutants in which the ZP domains were deleted (that is, DmZPC5-ΔC and DmZPC5-1/2ΔZP). These findings indicated that the acidic areas on the ZP protein surfaces were essential to the IMP activity of the proteins and that they were largely proportional to MPD activity.

## Discussion

ZP proteins are usually synthesized in developing oocytes as preproproteins and then secreted into the extracellular space^25^,^26^,^27^, where they form interconnected heterodimers after the removal of their C terminal at a furin cleavage site^16^,^17^. Fractions of ZP preproproteins are also found intracellularly in dense cored vesicles^28^,^29^,^30^,^31^. Upon fertilization, the extracellular ZP polymers are covalently cross-linked by transglutaminases that are secreted by the egg, which causes hardening of the chorion^32^. The ‘aggregation-prone' nature of the ZP proteins raises an obvious question: what tertiary structure is required for AnnotoZPs to exert IMP activity? We checked the polymerization status of three IMP-active proteins (DmZPAX1, DmZPC1 and DmZPC5) using non-denaturing polyacrylamide gel electrophoresis (PAGE)and found both ZP polymers and monomers are present in these preparations (Supplementary Fig. 8a). Occasionally, IMP-defective AnnotoZPs (for example, DmZPC5) were produced, either due to prolonged (>24 h) dialysis in water or as a result of an unknown cause. Interestingly, only the polymerized ZP forms were detected in these samples (Supplementary Fig. 8b). The chorion extracts from eggs of *T. bernacchii* and DmZPC5 transgenic zebrafish, which are active in IMP again contained the monomers and polymers, and the monomer fractions dramatically decreased after alkaline treatment with the loss of IMP (Supplementary Fig. 8b,c). To check whether hydrolyzation instead of polymerization of the ZP monomers had caused the loss of ZP monomers in the alkaline treated samples, we performed western blot analysis of DmZPC5 in alkaline-treated samples using denaturing SDS–PAGE (Supplementary Fig. 8d). DmZPC5 was found to be intact in all samples, without detecting any unnaturally low molecular weight products. We then examined the migration pattern of secreted DmZPC5 after 0.2 M alkaline treatment on a denaturing SDS–PAGE gel visualized by silver staining, again no significant level of degraded products were visible (Supplementary Fig. 8e). Taken together, it appears that the IMP activity of AnnotoZPs is conformation dependent and correlated with the amount of ZP monomers in a solution.

ZPs are well-described structural components of the chorion, the discovery of IMP activity in AnnotoZPs was unexpected. A concentration of 3 mg ml^−1^ of DmZPAX1 or DmZPC5 is equivalent to 29 and 50 μM, respectively, but the MPDs of these solutions were 0.65 and 0.56 °C (Table 1), exceeding the expected MPD values (0.00005 and 0.00009 °C, respectively) according to the colligative mechanism.

It is generally accepted that the mechanism underlying FP depression by AFPs and AFGPs is ‘adsorption-inhibition'^7^: by binding to a specific surface, AF(G)Ps create microcurvatures in the ice surface, and these inhibit ice growth via the Gibbs-Thomson effect^33^. However, in a recent study, Calvaresi *et al*.^34^ used molecular dynamics simulations of a type I AFP to show that this protein could induce local melting in ice or complete melting of nanoscopic ice crystals. Todde *et al*.^35^ reported that an AFP from Canadian snow flea triggered local melting of ice in many ice planes except the basal plane at temperatures below the MP of ice. Although further experimental evidence are needed to decipher the detail mechanisms for AFP-induced ice-melting, the results of these simulations pointed to important roles the water/ice interface has in the induced ice melting and implied that the binding of AFPs to the ice surface might be quasi-permanent or reversible^36^.

The clear presence of ice-faceting activity without significant thermal hysteresis in each of the AnnotoZPs found in this study is suggestive of reversible ZP–ice interaction. It is likely that AnnotoZPs facet ice crystals by adsorbing (or accumulating) at the ice/water interface, where they locally alter the electrostatic potential through the charged amino acids located in the acidic patches. The altered electrostatic potential weakens the hydrogen bonds between the water molecules on the surface of ice crystal, promoting dissociation of the affected water molecules from the ice surface when the temperature is elevated. IMP activity was nearly completely lost when we substituted the negatively charged residues of the ZP acidic patches, strongly suggesting the involvement of the electrostatic potential of the ZP proteins in ZP-induced accelerated ice-melting (Fig. 6f). The extended dynamic hydration shell of the AnnotoZPs might have crucial roles in this functional mechanism, as indicated by the fact that alkaline treatment of AnnotoZPs abolished their IMP activity (Supplementary Fig. 3c). The extended dynamic hydration shell has been proposed to be mandatory to the function of other ice-binding proteins, such as AFGP^37^ and a hyperactive AFP identified in an insect^38^. One point that needs to be further clarified is that the IMP activity of the AnnotoZPs does not indicate active energy consumption by AnnotoZPs. Instead, AnnotoZPs promoted dissociation of water molecules from ice surfaces through altered hydrogen-bonding dynamics, so that the affected ice surface melts at a faster rate than in solutions without AnnotoZPs when the same amount of heat (that is, energy) is applied, leading to the observed non-colligative MPD. The intrinsic mechanisms that underlie the ice-melting remain to be further explored.

Inhabiting a persistently freezing environment, the ability of Antarctic notothenioids to thrive relies not only on AFGPs, which protect adult fish from freezing, but also on mechanisms that prevent spawned eggs and hatchlings from freezing. The surface of the notothenioid egg chorion is similar to that of other teleosts in that it is penetrated by radical canals and a single micropyle^26^,^39^. Both of these openings are large enough for microscopic ice crystals to enter. Thus the eggs of Antarctic notothenioids may be vulnerable to freezing. Newly spawned eggs are likely protected by the presence of an adequate quantity of AFGPs^13^, as has been shown in *G. acuticpes,* in which the whole-egg homogenate displayed a thermal hysteresis of 1.02 °C, and a FP of −2.31°C (ref. 13). Interestingly, the corresponding MP was −1.29 °C, which was 0.23 °C lower than that of the adult serum (−1.06 °C), suggesting the presence of extra MP-depressing agents in the newly spawned eggs. The MP of the perivitelline fluid of the ready-to-hatch eggs reached −1.91 °C and the FP −1.97 °C, indicating that the level of inherited AFGPs diminished during hatching^13^. The source of the reagents that lowered the MP/FP at this stage was not defined. It cannot rule out the unpolymerized ZP proteins released into the perivitelline fluid from the yolk sac or the chorion contributed to MP/FP depression in newly spawned or ready-to-hatch eggs. In addition, high-latitude Antarctic notothenioid eggs may be in direct contact with or encased within surface ice^40^. An egg chorion that is fortified with agents promoting ice-melting is presumably beneficial for reducing the chance of ice penetration, which would improve the chance of survival. We estimated the concentration of AnnotoZPs in the chorion of developing eggs of *T. bernacchii*, and found the concentration is very likely greater than 5 mg ml^−1^. This concentration roughly matches or is greater than the concentrations (mostly 3 mg ml^−1^) we used for the MPD activity measurement in this study, suggesting presence of significant levels of AnnotoZP MPD activities *in vivo*. In addition IMP activity appears to be evolutionarily enhanced in Antarctic notothenioids, as was demonstrated by our finding that greater MPDs were caused by DmZPs than the orthologous proteins of the temperate species *E. maclovinus* (Supplementary Fig. 9).

A remarkable feature of AnnotoZP genes is the geo-specific distribution of large-scale duplications. The expansion of ZP genes parallels the evolutionary history of the notothenioids, and there is a significant correlation between ZP gene dosage and the severity of environmental temperatures (Fig. 2). The five notothenioids that currently inhabit the freezing continental shelf waters possess a large ZP gene family of ∼60 genes in *D. mawsoni* and a similar number in the other four species. *L. nudifrons*, *N. angustata* and *D. eleginoides*, which diverged from their respective Antarctic lineages and now permanently inhabit sub-Antarctic waters, possess intermediate numbers of ZP genes, with total ZP copy numbers significantly lower than those of their Antarctic cousins (Supplementary Table 2). The outgroup fish (*O. latipes*) and the two basal notothenioids contain only single gene for most of the ZP type (Supplementary Table 2). In *O. latipes* genome, the single-copy ZP orthologs are located in four linkage groups^41^, suggesting that the interspersed distribution of prototypic ZP genes is an ancient feature in teleosts. Given this fact, the remarkable multi-fold amplification of the seven AnnotoZP genes must have occurred repeatedly and independently at different chromosomal locations, suggesting that the function of the chorion in Antarctic notothenioid fishes has been under heavy selective pressure. Among the ZP types, ZPC5 and ZPAX1 are highly duplicated and abundantly expressed in *D. mawsoni* (Supplementary Table 2, Supplementary Fig. 1c). Coincidentally, these two types demonstrated the highest MPD activity (Table 1), lending another support to the notion that freezing temperatures have driven the expansion of ZP genes. On the other hand, the extent of expansion in each ZP type varies among the Antarctic species, suggesting species-specific patterns of ZP type duplication.

One major effect of low temperature is the slowing of all biochemical reactions. It is plausible that the demand for a sufficient quantity of ZP proteins within a limited developmental period has acted as one of the selection forces contributing to the increase in ZP gene copy number in Antarctic notothenioids. In fact, boosting protein production through gene duplication is a common evolutionary strategy when a larger quantity of a protein is favoured under certain environmental challenges^42^,^43^.

In addition to the substantial production of ZP proteins in the ovary, RNA-seq data revealed that AnnotoZPs are also expressed in other tissues, such as the testis, liver, brain, head kidney, skin and bones (Supplementary Fig. 1a). Ectopic expression of AnnotoZPs may suggest a need for MPD activity in other body compartments, which may have in turn facilitated the retention of duplicated ZP genes. A recent study reported that as a result of the effects of AFGP-induced melting inhibition, summer warming temperature might not reliably eliminate internal ice from Antarctic notothenioid body fluids^44^. It is possible that AnnotoZPs might be beneficial in reducing the body ice load. Indeed the ice-melting promoting ZPC5 monomers are present in the sera of Antarctic notothenioids (Supplementary Fig. 10). More direct evidence for the freezing-preventive role of AnnotoZPs came from the results of freezing resistance assays in the eggs of the DmZPC5 transgenic zebrafishes and the enhanced survival of zebrafish eggs when DmZPC5 was applied *in vitro* (Fig. 5). The improved survival achieved by a moderate level of an AnnotoZP supports the hypothesis that AnnotoZPs contribute to adaptation of the Antarctic notothenioids to the freezing environments.

In summary, AnnotoZPs have undergone extensive duplication under the selective pressure of freezing temperatures. *In vitro* and *in vivo* studies provide a body of evidence indicating functional co-option of a family of pre-existing ZP genes for freeze-prevention in Antarctic notothenioids. By characterizing the structural bases for the previously unrecognized IMP activity associated with AnnotoZPs, we reveal a new type of ice-binding macromolecule that function in a mechanism distinct to that of the known AFPs. Given the wide distribution of ZP proteins in the animal kingdom and the overall conservation of the ZP domain, it is likely that evolution toward enhanced ice-melting activity may also have occurred in the ZP proteins of other polar organisms that have experienced similar selective pressures.

## Methods

### Specimen and tissue collection

Notothenioid species were collected by line fishing or trawling at the locations listed in Supplementary Table 3. All tissues were flash frozen in liquid nitrogen and stored at −80 °C until use. The animal sampling and experimental protocol was approved by the Ethics Committee for the Use of Animal Subjects of Shanghai Ocean University.

### *D. mawsoni* ZP cDNA characterization

ZP domain-containing ESTs of *D. mawsoni* were obtained from GenBank and assembled using CAP3 (ref. 45). The unigenes were sub-typed based on the best-matched ZP types in *O. latipes*^41^. To obtain the full-length coding sequences, respective cDNA clones were identified from a *D. mawsoni* ovary cDNA library and sequenced to completion.

### Characterization of ZP genes in other notothenioid species

RNA was purified from tissues of four notothenioid species, including *T. bernacchii*, *G. acuticeps*, *C. hamatus* and *E. maclovinus*, using a standard Trizol protocol. After quality assessment, three micrograms of high quality total RNA from each sample was used to prepare an mRNA-Seq library with a TruSeq RNA Sample Prep Kit (Illumina) according to the manufacturer's instructions. Qualified libraries with an insert size of 300–400 bp were subjected to 2 × 100-bp paired-end sequencing using HiSeq 1500 (Illumina). The raw Illumina reads were trimmed to a Q score greater than 30 and assembled using the Trinity package^46^,^47^. The assembled transcripts were annotated using BLASTX against the current Nr database with the E-value set at 1E-20. Transcripts containing ZP domains were collected for further analyses.

We designed primers based on the RNA sequences of the AnnotoZPs, and used for reverse transcriptase–PCR to amplify the full-length coding sequences of desired AnnotoZPs from ovary RNA of the species. The PCR products were cloned into the pMD-19T vector (Takara) and sequenced.

### Real-time-PCR quantification of DmZP gene expression

*D. mawsoni* ovary total RNA was reverse-transcribed using M-MLV superscript reverse transcriptase according to the manufacturer's instructions. Real-time PCR was performed using the SYBR Premix Ex TaqTM (DRR041A, Takara Bio) and a LightCycler 480 PCR machine (Roche). The primers used are listed in Supplementary Table 4. The mRNA expression levels of each gene were normalized to the level of the housekeeping gene β-actin.

### Determination of ZP copy number variation in fish genomes

To determine the copy numbers of the annotoZPs in fish genomes, we performed absolute quantitative PCR (qPCR) using a FastStart Universal SYBR Green Master Kit (Roche Applied Science) according to the manufacturer's instructions. We used the most conserved regions of eight ZP genes (ZPC1, ZPC2, ZPC3, ZPC4, ZPC5, ZPB, ZPAX1 and ZPAX2) to design species-specific qPCR primers (Supplementary Table 4). The target DNA fragment of each gene was cloned and sequenced to verify its identity. To construct standard amplification curve, plasmids containing the target ZP fragments were linearized and purified using a QIA-quick gel extraction kit (QIAGEN), and concentrations were determined using a Nanodrop 2000C spectrophotometer (Thermo Scientific). The corresponding copy numbers of the ZP plasmids were calculated using the following equation^48^: DNA (copy)=6.02 × 10^23^ (copy per mol) × DNA amount (g)/DNA length (bp) × 660 (g mol^−1^ per bp). Serially diluted ZP plasmids of known concentrations were used to construct standard curves. Genomic DNAs from notothenioids were prepared using phenol/chloroform extraction and ethanol precipitation, and concentrations were determined. Real-time PCR was prepared in a final volume of 20 μl containing 10 μl of SYBR Premix Ex TaqTM, 1 μl of primer mix (0.5 μM of each primer), 8 μl of H_2_O and 1 μl of genomic DNA (50–100 ng μl^−1^) or the standard DNA in a LightCycler capillary tube. PCR was performed at 95 °C for 10 min followed by amplification for 40 cycles at 95 °C for 10 s and 60 °C for 15 s using a LightCycler 480 Real-Time PCR System (Roche). A melting curve analysis was performed according to the manufacturer's instructions (Roche). The C-values for *D. mawsoni*, *N. coriiceps*, *G. acuticeps*, *C. hamatus*, *T. bernacchii*, *N. angustata*, *L. nudifrons* and *E. maclovinus* were found in the literatures^19^,^43^,^49^,^50^, as follows: 1.20, 1.13, 1.34, 1.79, 1.19, 1.315, 0.99 and 1 pg, respectively^51^. One nanogram of genomic DNA from *D. mawsoni*, *N. coriiceps*, *G. acuticeps*, *C. hamatus*, *T.bernacchii*, *N. angustata*, *D. eleginoides*, *L. nudifrons* and *E. maclovinus* was estimated to contain 833, 885, 746, 559, 840, 760, 833, 1,000 and 1,000 copies of haploid genome, respectively. The gene copy number per haploid genome was then calculated as the gene copies per ng divided by the copies of the haploid genome per nanogram. Each qPCR assay was repeated at least in triplicates and the technical variations between each group were similar.

### Expression vector construction

The full-length AnnotoZP sequences were engineered to contain a FLAG octapeptide and cloned into the expression vector pIRES2-EGFP (Supplementary Fig. 2c). DmZPC5 domains were serially deleted using PCR amplification of the DmZPC5 expression vector with primers designed to eliminate desired coding sequences. The PCR products were then recircularized.

A set of gene segments encoding site-directed variants of DmZPC5^ΔA/B^ containing 17 substitutions (D88N, D97N, D99N, E153Q, D163N, E164Q, D288N, D289N, E303Q, D304N, E307Q, E317N, D321N, D323N, D340N, D343N and E354Q) was chemically synthesized (Sangon Biotech), subcloned into an EcoRI/SmaI-digested pIRES2-EGFP plasmid and verified by sequencing. A fragment containing the D88N, D97N, D99N, E153Q, D163N and E164Q substitutions was excised from DmZPC5^ΔA/B^ by EcoRI and SalI double digestion, and used to replace the homologous sequence within the pIRES2-DmZPC5 clone to generate the DmZPC5^ΔA1^ mutant. Similarly, the fragment containing the D288N, D289N, E303Q, D304N, E307Q, E317N, D321N, D323N, D340N, D343N and E354Q substitutions was obtained from DmZPC5^ΔA/B^ by SalI/SmaI double digestion and used to replace the homologous sequence in pIRES2-DmZPC5 to generate the DmZPC5^ΔA2/B^ clone. The detailed sequences are shown in Supplementary Fig. 5.

### Protein expression, purification and solute content control

CHO cells (American Type Culture Collection CCL-61) were obtained from American Type Culture Collection and cultured in Dulbecco's modified Eagle's medium containing 10% fetal bovine serum (Gibco). CHO cell cultures were tested negative for mycoplasm. At 70–90% confluence, the cells were transfected with the respective expression vectors using the TurboFect transfection reagent (Thermo) and the cultural medium was replaced with the conditioned medium (Freestyle CHO Expression Medium). After 48 h, adherent cells (∼0.7–1.1 × 10^6^) were washed once with cold PBS, and lysed using 1 ml mammalian cell lysis/extraction reagent (Sigma, added 1% Triton X-100 and 1% protease inhibitor Cocktail) by incubating the plate on ice for 30 min. The lysate was collected and centrifuged at 12,000*g* for 10 min, and the supernatant was recovered and placed into a fresh tube. To isolate the FLAG-tagged AnnotoZPs, 100 μl of ANTI-FLAG M2 affinity resin (Sigma) was added to 1 ml of lysate (or the culture medium for secreted AnnotoZPs). The protein-antibody mixture was kept at 4 °C overnight with gentle rotation. The mixture was then centrifuged for 30 s at 6,000*g*, and the supernatant was removed. The pellet was then washed with 1 × TBS three times. To elute the protein, 100 μl of 3 × FLAG peptide (150 ng μl^−1^, Sigma) was added, followed by incubation at 2–8 °C for 30 min and centrifugation for 30 s at 5,000*g* and the supernatant containing the eluted ZP was recovered. The protein eluate (∼200 μl) was loaded to a Spin Desalting Column (Zeba), centrifuged gently to eliminate salt and molecules smaller than 7 kD. The protein concentration was determined by absorbance measurements at 280 nm using a Nanodrop 2,000 (Thermo). Ion chromatography ICS900 (cation) and ICS1500 (anion) (Thermo) or a vapor pressure osmometer (Wescor Inc, Utah, USA) were used to measure ion levels or osmolarity of the prepared samples to assure the absence of unwanted solute contamination. The purity and the polymerization status of the purified ZPs were checked by denaturing or non-denaturing PAGE gels, respectively. ZP proteins with good quality were concentrated by lyophilization and resuspended in 1 × TBS for desired concentrations.

### Ice crystal morphology and FP/MP measurements

The morphologies of ice crystals were monitored during growth and melting using an Otago Nanoliter Osmometer (Otago Osmometers Ltd., Dunedin, New Zealand) according to the manufacturer's instructions. Before the samples were measured, the device was calibrated using distilled water and 6% ethanol to obtain the ‘zero' and ‘−2 °C' points, respectively. To measure the FP of a sample, a drop of sample that was ∼1 mm in diameter (∼0.5 nl) was injected into an immersion oil B-filled sample well, placed on a temperature-controlled stage and observed under a microscope. The temperature close to the sample was determined with a precision of 0.01 °C. Initially, the samples in the wells were quickly frozen. The device was then slowly warmed to allow slow melting of the frozen sample until only a single ice crystal remained. The temperature at which the last ice crystal melted was taken to indicate the MP. The sample was frozen and re-melted to obtain a single ice crystal of ∼10 μm in diameter. After stabilizing the ice for 5 min, the device was cooled, and the ice growth behaviour was closely monitored. The temperature at which the crystal began to grow was taken to indicate the freezing point. The crystal was then warmed and re-cooled to the desired temperature several times to inspect the shape of the crystal. To ensure data consistency, each sample was evaluated at least three times using protein preparations obtained from different batches of transfections and purifications.

### Measurement of MP in alkaline-treated ZP solutions

Two aliquots containing an equal volume (2 μl) were taken from each ZP sample (∼6 mg ml^−1^ in 1 × TBS) for testing. A volume of 2 μl of 0.4 M NaOH (dissolved in water) was added to one aliquot, and an equal volume (2 μl) of 1 × TBS was added to another aliquot. The MPs and FPs of the NaOH-treated and the native samples were measured using the method described above.

### Construction of DmZPC5 transgenic zebrafish lines

We constructed a Tol2 transposable element-based transgenic vector containing a 524- bp DrZP3 promoter^24^, a multiple cloning sequence followed by IRES and the EGFP sequence flanked by the arms of the Tol2 transposon. The DmZPC5 cDNA was cloned into this vector. A total of 50 ng μl^−1^ of the transgene construct was mixed with 50 ngμl ^−1^ of the transposase mRNA and injected into single-cell zebrafish embryos. Two female founders were generated, and these were mated with wild-type males. Transgene-positive F1 fishes were raised to sexual maturity for egg collection and analysis.

### Extraction of chorion proteins

Notothenioid oocytes were collected from frozen ovary tissues by dissolving and rinsing the tissues thoroughly in 1 × TBS. For zebrafish and other species used in this study, gravid female fishes were anesthetized in tricaine and placed in a plastic petri dish. The belly of the fish was squeezed to obtain eggs. The eggs were rinsed thoroughly with 1 × TBS, and the contents were removed by squeezing the oocytes between two nylon mesh membranes. The eggshells were recovered and rinsed in a large volume of solution (100 mM EDTA, 300 mM NaCl, 0.1% phenylmethylsulphonyl fluoride, pH 7.2) for three times with 1 min of centrifugation (1,500*g* at 4 °C) between each wash step. The supernatant was removed, and the eggshells were homogenized using a microcentrifuge tube homogenizer in 1 × TNE buffer (50 mM Tris HC1, 125 mM NaC1, 10 mM EDTA, pH 7.2) containing 0.1% Triton X-100 and 1 mM phenylmethylsulphonyl fluoride on ice. The homogenate was then centrifuged at 13,000*g* for 5 min at 4 °C and supernatant was recovered. Appropriate volume of 1 × TNE was added to make the desired concentrations.

### Immunohistochemical analysis of transgene expression

Rabbit polyclonal antibodies against the N-terminal 284 amino acids of DmZPC5 were custom made by HuaAn Biotechnology Co., Ltd. (Hangzhou, China).

After euthanized, the abdominal cavity of a zebrafish was surgically exposed to obtain the ovaries. The ovaries were immersed in 4% paraformaldehyde fixative overnight at 4 °C and embedded in paraffin. The tissues were sectioned (8 μm), deparaffinized, and rehydrated in a graded series of ethanol solutions and washed 2 × 5 min in PBS. The ovary sections were then treated with 10 mM sodium citrate (pH 6.0) at a sub-boiling temperature for 10 min. The sections were incubated at room temperature for 10 min in 3% (v/v in methanol) hydrogen peroxide to block endogenous peroxidase activity. The sections were then blocked in TNK buffer (0.1 M Tris, 0.55 M NaCl, 0.1 mM KCl, 1% goat serum, 0.5% BSA, and 0.1% Triton X-100 in PBS) at room temperature for 1 h. After wash in PBS, the sections were sequentially incubated in primary and secondary antibody solutions. The sections were then incubated with 3,3′-diaminobenzidine tetrahydrochloride (DAB) solution for 30 min at room temperature to visualize the signal, counterstained, dehydrated and mounted. Images were photographed using a Zeiss microscope equipped with an AxioCamMRc5.

### Western blot analysis of ZP proteins.

#### Denaturing SDS–PAGE analysis

We cast 10% SDS–PAGE gel using 10% acrylamide/bisacrylamide (29:1), 0.1% SDS, 350 mM Tris-HCl (pH 8.8); the gels were polymerized with 0.1% ammonium persulfate and 4 μl N, N, N′, N′-tetramethylethylenediamine (TEMED). Before protein loading, denaturing loading buffer (50 mM dithiothreitol, 1% SDS, 10% glycerol in 10 mM Tris-HCl (pH8.0)) was added and boiled for 10 min. Electrophoresis was performed at 100 v for 90 min in a buffer containing glycine (193 mM), tris-base (25 mM) and 0.1% SDS.

#### Non-denaturing PAGE analysis

The PAGE gel formula was similar to that of denaturing gel, except that no SDS was added. The protein-loading buffer contained no SDS or dithiothreitol, and the protein sample was not boiled before loading. The electrophoresis buffer contained 193 mM glycine and 25 mM Tris-base with no SDS.

After electrophoresis, the proteins were transferred to a nitrocellulose membrane (Millipore) using electrotransfer (mini-protein Tetra System, BioRad) in a buffer solution containing 193 mM glycine, 25 mM Tris (pH 8.3) and 20% methanol. The membrane was treated with blocking agent (5% nonfat milk in 1x TBST) for 2 h at room temperature on a shaking bed. Anti-DmZPC5 antibody was added, and the membranes were incubated at room temperature for 1 h. The membrane was then washed with 1 × TBST three times for 15 min each. The secondary antibody (1:2,000 in 1 × TBST, Boston Biomedical Inc.) was then added and incubated for 1 h at room temperature. The membrane was washed with 1 × TBST twice and 1 × TBS once for 15 min each. Colour was developed using SuperSignal West Pico Chemiluminescent Substrate (Thermo) according to the manufacturer's instructions. Images were acquired using a ChemiDoc MP Imaging System (BioRad).

### Freezing resistance assay of fertilized zebrafish eggs

Fertilized eggs were collected from the wild-type zebrafishes and from the mating pairs of DmZPC5^+/−^ transgenic fishes, respectively. The collected eggs were cultured at 28 °C for 3 h in E3 hatch medium (0.29 g NaCl, 0.013 g KCl, 0.036 g CaCl_2_·2H_2_O, 0.06 g MgSO_4_·6H_2_O and 0.25 mg methylene blue in 1 litre) and the non-developing eggs were removed. About 80 normally developing eggs from each group were separately transferred to a glass tube with 2 ml of E3 medium and submerged to a glycol-water bath for 20 min, which was stably controlled at −2 °C. The treated eggs were then incubated at 28 °C. After 4–5 h. The eggs were examined under a light microscope, the whitened eggs (for example, dead eggs) and the normally developing (survival) eggs were counted. This test were repeated multiple times with different batches of eggs.

To further verify the effect of AnnotoZPs on egg survival, culture medium of CHO cells which express DmZPC5 was applied to wild-type zebrafish eggs and the survival rate at a mild freezing temperature (0 °C) was determined. Briefly, the *DmZPC5* expressing vector and the empty vector (Supplementary Fig. 2c) were transfected to CHO cells, respectively, as described in the ‘Protein expression' Section above. After 4 days of culturing in the Freestyle CHO Expression Medium, the culture medium (about 30 ml) was collected and centrifuged at 12,000*g* for 10 min at 4 °C to remove cell debris. The supernatant was transferred to a Amicon Ultra-4 centrifugal filter units (Millipore, #UFC801096) to concentrate proteins. The retained medium (10 ml) was dialysed against the E3 hatch medium overnight at 4 °C and the protein content was visualized in a SDS–PAGE gel followed with western blot verification. The protein concentration was measured using the BCA Protein Assay Kit (Pierce). A concentration of ∼5 mg ml^−1^ of the ZP protein was included in the medium. The culture medium of CHO cells transfected with the empty vector (expresses an EGFP) was prepared using the same protocol and used as background control in freezing resistance assays.

Naturally fertilized zebrafish eggs were collected timely and incubated in hatch medium at 28 °C for ∼3.5 h. The unfertilized eggs were removed. Normally developing eggs from different mating pairs were pooled and equally divided to ensure homogeneity of egg quality for each assay. The hatch medium was removed and 2 ml of the ZP containing medium and the control medium were separately added to wells of a 6-well plate, each containing about 60 eggs. The eggs were subjected to 0 °C for 40 min and then moved to 28 °C for further development. The survival and dead embryos were counted after 5 h. The assay was repeated three times with eggs from different parents and proteins prepared from different batches.

### Homology-based molecular structure modelling

The three-dimensional structures of Dm-ZPC5 and its mutation variants were modelled in the on-line automated Build Homology Model program at Phyre2 (ref. 42) using the crystal structure of ZP 3 from Gallus gallus (PDB accession code 3NK4, resolution 2.0 Å) as the template. The generated structures were analysed and verified using the Structural Analysis and Verification Server (http://services.mbi.ucla.edu/SAVES/)^52^,^53^. Structures were visualized using PyMOL (http://www.pymol.org/)^54^.

### Statistical analyses

All experiments were repeated a minimum of three times. Data are expressed as mean±s.d. The main statistical test was the unpaired Student's *t*-test. For experiments involving multiple comparisons, we used one-way analysis of variance tests or *post hoc* comparisons (Tukey's) tests. All the statistical analyses were performed with Prism software. *P* values less than 0.05 were considered to be statistically significant.

### Data availability

GenBank accession numbers for the previously determined DNA sequences analysed in the current study are shown in Supplementary Table 1. The authors declare that the data supporting the findings of this study are included within the article and its Supplementary information files, or are available from the authors on request.

## Additional information

**How to cite this article:** Cao, L. *et al*. Neofunctionalization of zona pellucida proteins enhances freeze-prevention in the eggs of Antarctic notothenioids. *Nat. Commun.* **7**, 12987 doi: 10.1038/ncomms12987 (2016).

## Supplementary Material

Supplementary Information Supplementary Figures 1-10, Supplementary Tables 1-4 and Supplementary References

## Figures and Tables

**Table 1:** Freezing/melting point depression by recombinant *D. mawsoni* ZPs.

	Melting point depression (°C)	Freezing point depression (°C)	Thermal hysteresis (°C)
DmZPAX1	0.650±0.17	0.662±0.17	0.010±0.00
DmZPC5	0.560±0.05	0.572±0.05	0.012±0.01
DmZPD	0.347±0.02	0.360±0.01	0.013±0.01
DmZPB	0.287±0.01	0.310±0.01	0.023±0.01
DmZPC1	0.257±0.02	0.283±0.02	0.027±0.02
BSA	0.007±0.02	0.010±0.03	0.010±0.00
