# Supplementary material for: Neofunctionalization of zona pellucida proteins enhances freeze-prevention in the eggs of Antarctic notothenioids
Source: Nat Commun. 2016 Oct 4;7:12987. doi: 10.1038/ncomms12987 (PMC5059455; doi:10.1038/ncomms12987)
Supplement: Supplementary Information — Supplementary Figures 1-10, Supplementary Tables 1-4 and Supplementary References [file ncomms12987-s1.pdf]

Supplementary Information

Containing 10 supplementary figures and 4 supplementary tables

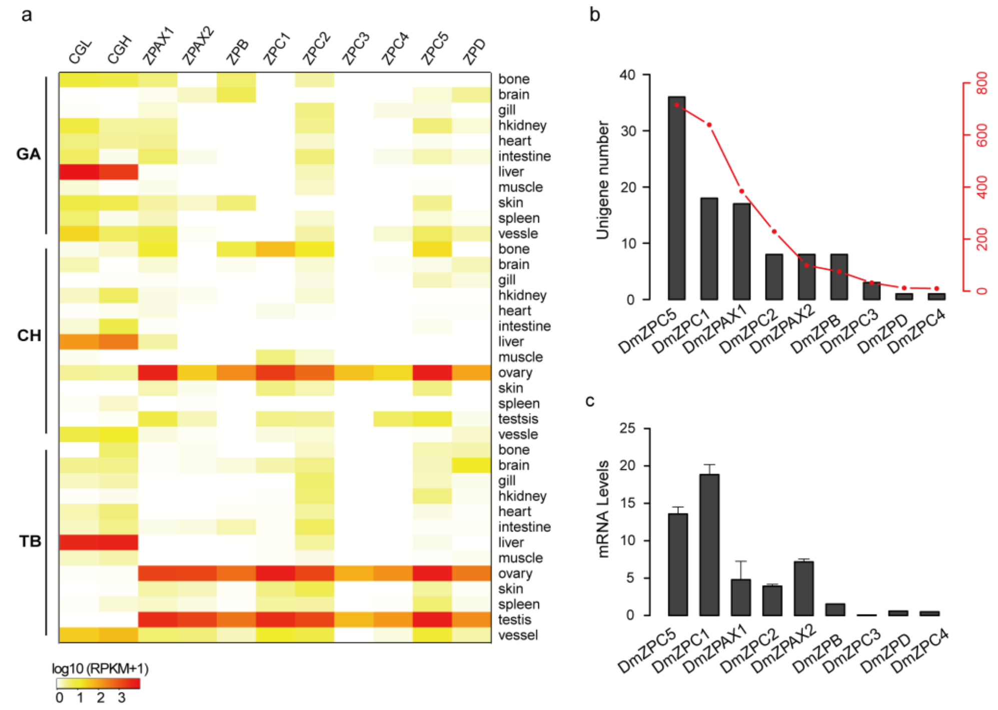

Supplementary Figure 1. (a). Heatmap showing the ubiquitous expression of ZP gene family members in multiple tissues in three Antarctic notothenioids (*G. acuticeps, C. hamatus,* and *T. bernacchii*). Gene expression levels were calculated in 11 tissues using RPKM values that were deduced using RNA-seq. The 11 ZP genes analyzed were shown on the top. The tissues examined in these species are shown to the right. The color scales represent the log 10 transformations of expression levels (RPKM). The RNA-seq data of *T. bernacchii* are deposited to the NCBI GenBank under the Accession no. SRP068525, and the head kidney for C. hamatus and G. acuticeps are deposited to NCBI Genbank under the accessions no. GSE70113 through another published paper (Xu et al., 2016). **(b). Transcript abundance of the ZP types deduced from sequencing of *D. mawsoni* ovary and liver cDNA libraries (Chen et al., 2008).** The gray bar represents the unigene numbers of each ZP type, and the dotted line represents the total number of ESTs of each type that were found in the two transcriptomes. **(c). The expression levels of the ZP genes in *D. mawsoni* ovary tissues were detected using real-time PCR (normalized to actin, shown as mean with SD, n=3).**

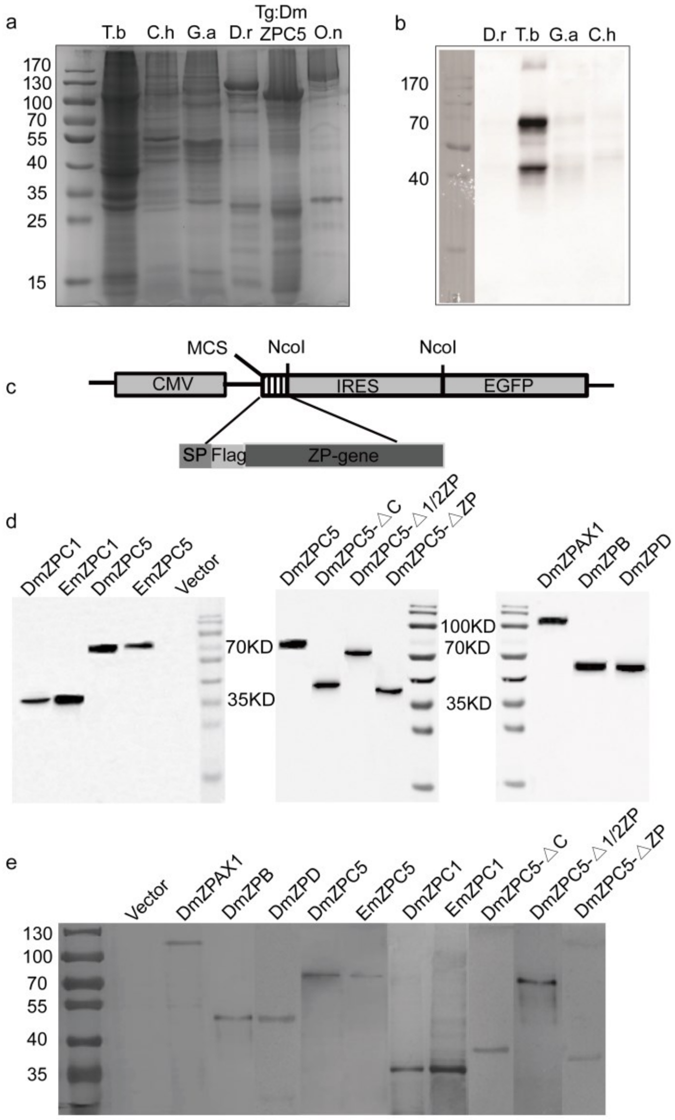

Supplementary Figure 2. Chorion protein extractions and recombinant expression of the AnnotoZPs. (a). SDS-PAGE (denaturing) gel showing the complexity of the extracted chorion proteins obtained from *T. bernacchii*, *C. hamatus*, *G. acuticeps*, *D. rerio*, transgenic zebrafish and *O. niloticus*. Approximately 12-20 μg of total proteins were loaded on a 10% SDS-PAGE. Silver staining was used to visualize the protein bands. The extracted chorion proteins were heterogeneous in size as a result of complex polymerization schemes between the various ZP types. This may also have resulted from the presence of non-ZP proteins in the chorion. **(b). Western blot analysis of egg chorion proteins obtained from four fish species were separated by SDS-PAGE and labeled with the anti-dmZPC5 antibody**. ZPC5 was identified in Antarctic fish chorions in two forms with different sizes: a C-terminal cleaved form (a 45 kD band) and an uncleaved preproprotein (a 75 kD band). The 75 kD isoform indicates that a large

percentage of ZPC5 is not processed *in vivo*. A fraction of ZPC5 remained in a polymerized state (shown as a band that was bigger than 170 kD) as a result of incomplete dissociation under electrophoretic conditions. The anti-DmZPC5 antibody interacted poorly with the zebrafish ZPs (lane D.r). **(c). The vector and the cloning scheme used for recombinant expression of AnnotoZPs**. **(d).** Western blot analysis was performed using an anti-FLAG antibody to probe the product of the recombinant AnnotoZPs. A total of 50 μg of crude protein was extracted from the transfected CHO cells and loaded for 10% SDS-PAGE. The 'vector' lane contains the protein that was extracted from the cells that were transfected with the empty vector, and the other lanes are labeled to indicate each of the recombinant ZP types that were expressed. **(e). Examination of the quality and the quantity of the affinity chromatography-purified recombinant proteins.** Approximately 5 μg of each protein was loaded onto SDS-PAGE gels and visualized using silver staining.

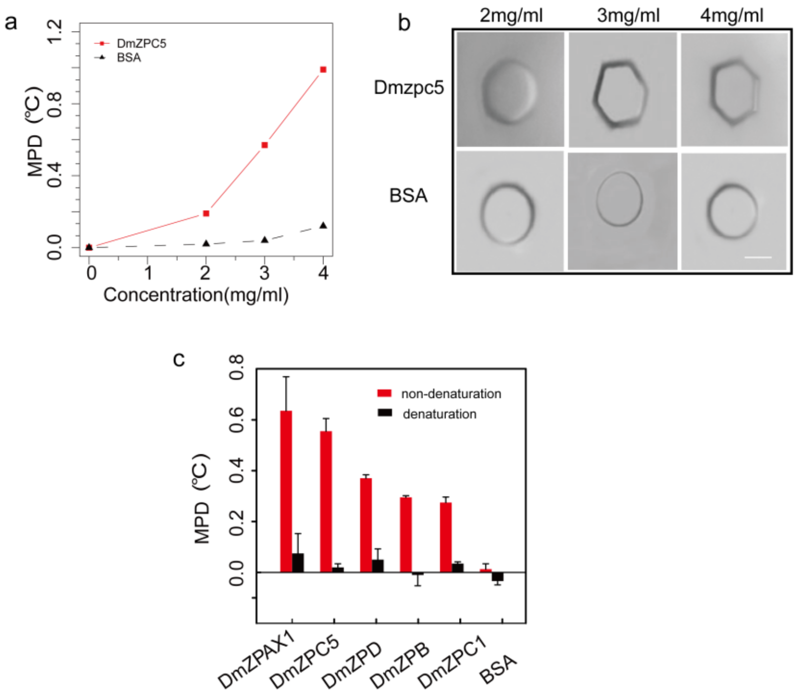

Supplementary Figure 3. The non-colligative melting point depression induced by the recombinant AnnotoZPs. (a) The relationship between melting point depression and the concentration of ZPC5 and comparison with a control protein, BSA (bovine serum albumin).

(b). The morphology of the ice crystals that were grown at different protein concentrations. The freezing and melting point data were measured using a 1x TBS solution. **(c). The melting point depression (MPD) activity of AnnotoZPs was significantly reduced by alkaline-denaturation.** Each protein sample intended for the denaturing test was divided into two aliquots (2 µl each). Equal volumes (2 µl each) of either 0.4 M NaOH (dissolved in water) or 1x TBS was added to each aliquot. After incubating the solutions for 10 min at room temperature, the melting and freezing points of the treated (denatured with NaOH) and native (non-denatured in 1x TBS) samples were measured using a Nanoliter Osmometer. The protein concentrations were 3 mg/ml in each sample. Melting point depression was greatly reduced in the AnnotoZP samples, while no change was observed (both were close to 0) for BSA.

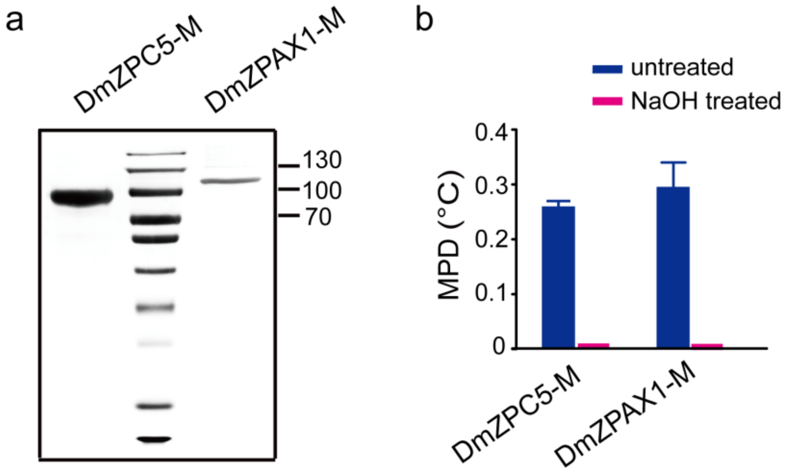

Supplementary Figure 4. MPD activity assay for secreted DmZPC5 and DmZPAX1. (a). PAGE analysis of secreted DmZPC5 and DmZPAX1 (labeled as DmZPC5-M, DmZPAX1-M respectively) purified from the culture media of CHO cells expressing the ZP proteins. The proteins were visualized by silver staining. (b) MPD activities measured from the two secreted proteins in the native and NaOH-treated samples.

```
                                          flag
DmZPC5△A1      MEAFNFQVILLVGLCVSSSFA DYKDDDDK FPPTRYTQDASFQSLANTSRSEIVQQQQQQK
DmZPC5△A/B     MEAFNFQVILLVGLCVSSSFA DYKDDDDK FPPTRYTQDASFQSLANTSRSEIVQQQQQQK
DmZPC5△A2/B    MEAFNFQVILLVGLCVSSSFA DYKDDDDK FPPTRYTQDASFQSLANTSRSEIVQQQQQQK
DmZPC5         MEAFNFQVILLVGLCVSSSFA DYKDDDDK FPPTRYTQDASFQSLANTSRSEIVQQQQQQK
               ********************* ******** *******************************

DmZPC5△A1      SKAEEPQQVNTIRV TCHPDSLEIVIKANMFAVGAPVNANEIRLGVETNNQYCRATASSAD
DmZPC5△A/B     SKAEEPQQVNTIRV TCHPDSLEIVIKANMFAVGAPVNANEIRLGVETNNQYCRATASSAD
DmZPC5△A2/B    SKAEEPQQVNTIRV TCHPDSLEIVIKADMFAVGAPVDADEIRLGVETNNQYCRATASSAD
DmZPC5         SKAEEPQQVNTIRV TCHPDSLEIVIKADMFAVGAPVDADEIRLGVETNNQYCRATASSAD
               ************** ************* ******** * *********************

DmZPC5△A1      EYSISVGLVECGTRHWVTEDSLIYTNLLIYSPQASPYGVVRMNQAVIPIECHYERKYSVS
DmZPC5△A/B     EYSISVGLVECGTRHWVTEDSLIYTNLLIYSPQASPYGVVRMNQAVIPIECHYERKYSVS
DmZPC5△A2/B    EYSISVGLVECGTRHWVTEDSLIYTNLLIYSPEASPYGVVRMDEAVIPIECHYERKYSVS
DmZPC5         EYSISVGLVECGTRHWVTEDSLIYTNLLIYSPEASPYGVVRMDEAVIPIECHYERKYSVS
               ********************************.********* .****************
                                       zp domain
DmZPC5△A1      SSSLMPTWIPFMSTQAAVEMLQFNLRIMTSDWQYKRSSNVFHLGEPISIEASVRIGHHMG
DmZPC5△A/B     SSSLMPTWIPFMSTQAAVEMLQFNLRIMTSDWQYKRSSNVFHLGEPISIEASVRIGHHMG
DmZPC5△A2/B    SSSLMPTWIPFMSTQAAVEMLQFNLRIMTSDWQYKRSSNVFHLGEPISIEASVRIGHHMG
DmZPC5         SSSLMPTWIPFMSTQAAVEMLQFNLRIMTSDWQYKRSSNVFHLGEPISIEASVRIGHHMG
               ************************************************************

DmZPC5△A1      LRVFVSSCVATLSPDMNSSPRHAFIENGCFVDSQLPGSRSQFLARTQDDKLHMSIDAFRF
DmZPC5△A/B     LRVFVSSCVATLSPDMNSSPRHAFIENGCFVDSQLPGSRSQFLARTQNNKLHMSIDAFRF
DmZPC5△A2/B    LRVFVSSCVATLSPDMNSSPRHAFIENGCFVDSQLPGSRSQFLARTQNNKLHMSIDAFRF
DmZPC5         LRVFVSSCVATLSPDMNSSPRHAFIENGCFVDSQLPGSRSQFLARTQDDKLHMSIDAFRF
               ***********************************************  ***********

DmZPC5△A1      YNEDRGELYITCHLNAEPINDADATNKACTFV NGRWRSADGNDYLCGQCKRSIEVEQTPS
DmZPC5△A/B     YNQNRGQLYITCHLNAQPINNANATNKACTFV NGRWRSANGNNYLCGQCKRSIQVEQTPS
DmZPC5△A2/B    YNQNRGQLYITCHLNAQPINNANATNKACTFV NGRWRSANGNNYLCGQCKRSIQVEQTPS
DmZPC5         YNEDRGELYITCHLNAEPINDADATNKACTFV NGRWRSADGNDYLCGQCKRSIEVEQTPS
               **. **.*********.*** * ********* ******* ** **********.******

DmZPC5△A1      KPSSPSKFRPRGFVKPEEREPLWRSGLKTSTVWEHQARVGPLMVLPAKQKSRPIPAKQRS
DmZPC5△A/B     KPSSPSKFRPRGFVKPEEREPLWRSGLKTSTVWEHQARVGPLMVLPAKQKSRPIPAKQRS
DmZPC5△A2/B    KPSSPSKFRPRGFVKPEEREPLWRSGLKTSTVWEHQARVGPLMVLPAKQKSRPIPAKQRS
DmZPC5         KPSSPSKFRPRGFVKPEEREPLWRSGLKTSTVWEHQARVGPLMVLPAKQKSRPIPAKQRS
               ************************************************************
```

DmZPC5$^{\Delta A1}$	SILDQISRSTMYGSQWRSGINRVDQRKGLLPDSSSTQNQVAVLTLASEQNQDGEDKSGTE
DmZPC5$^{\Delta A/B}$	SILDQISRSTMYGSQWRSGINRVDQRKGLLPDSSSTQNQVAVLTLASEQNQDGEDKSGTE
DmZPC5$^{\Delta A2/B}$	SILDQISRSTMYGSQWRSGINRVDQRKGLLPDSSSTQNQVAVLTLASEQNQDGEDKSGTE
DmZPC5	SILDQISRSTMYGSQWRSGINRVDQRKGLLPDSSSTQNQVAVLTLASEQNQDGEDKSGTE
	**
DmZPC5$^{\Delta A1}$	KDEDAEEVHELLEKTSPEAHLQSKAAVLNGTDTAALDEVFPTAAVNVAVPPLSNTTATVS
DmZPC5$^{\Delta A/B}$	KDEDAEEVHELLEKTSPEAHLQSKAAVLNGTDTAALDEVFPTAAVNVAVPPLSNTTATVS
DmZPC5$^{\Delta A2/B}$	KDEDAEEVHELLEKTSPEAHLQSKAAVLNGTDTAALDEVFPTAAVNVAVPPLSNTTATVS
DmZPC5	KDEDAEEVHELLEKTSPEAHLQSKAAVLNGTDTAALDEVFPTAAVNVAVPPLSNTTATVS
	**
DmZPC5$^{\Delta A1}$	DLSETMDPKRK
DmZPC5$^{\Delta A/B}$	DLSETMDPKRK
DmZPC5$^{\Delta A2/B}$	DLSETMDPKRK
DmZPC5	DLSETMDPKRK
	***********

Supplementary Figure 5. Amino acid alignment of DmZPC5 and site-mutated variants showing the identity of the amino acids that comprise the acidic patches on the surface of native DmZPC5 and the detailed amino acid substitutions in the site-mutated DmZPC5s (DmZPC5$^{\Delta A1}$, DmZPC5$^{\Delta A2/B}$ and DmZPC5$^{\Delta A/B}$). The amino acids located within the ZP domains were boxed in a red rectangle. The Glutamine (Q) and Asparagine (N) that are used to replace the negatively charged amino acids - Glutamic acid（E）and aspartic acid (D), which constitute the predicted acid patches were shown in color shadows.

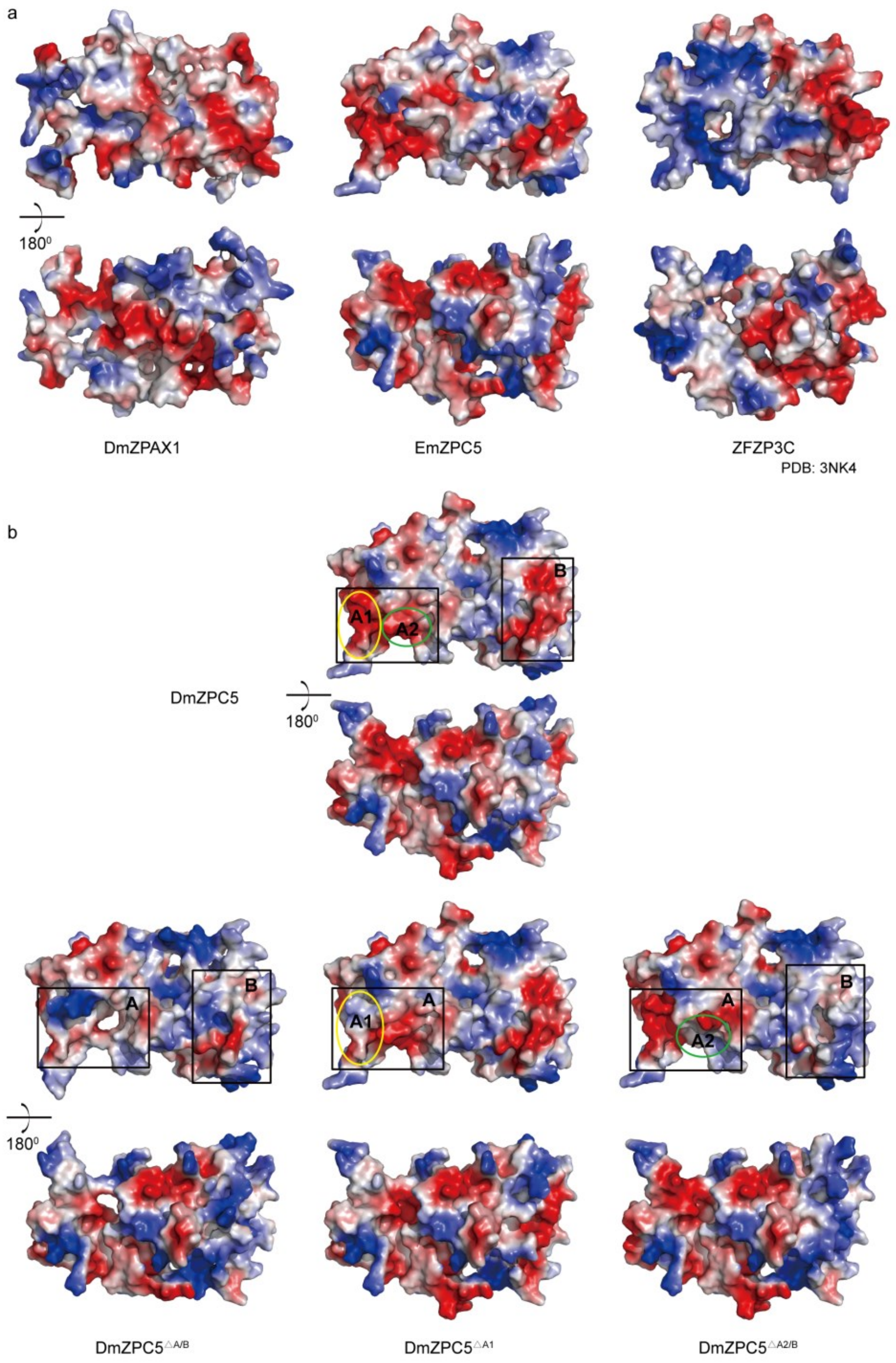

Supplementary Figure 6. The distribution of acidic amino acid patches on the surfaces of native and site-mutated ZP proteins was revealed by structure modeling analyses. (a) The electrostatic surface potentials of DmZPAX1, EmZPC5 and ZFZP3C were compared. Larger areas of acidic patches (shown in red) were observed in DmZPAX1 and EmZPC5 than in ZFZP3C. (b) Schematic 3-D models showing the distribution of basic and acidic regions on the surfaces of the three site-directed mutants of DmZPC5 (DmZPC5$^{\triangle A/B}$, DmZPC5$^{\triangle A1/B}$, and DmZPC5$^{\triangle A2/B}$). The

areas were smaller than those in native DmZPC5. The acidic-A and -B regions are marked in rectangular boxes. The subareas corresponding to the acidic-A region are marked with oval circles. Blue indicates basic (positively charged) regions, and red indicates acidic (negatively charge) regions. Color intensity reflects the charge intensity.

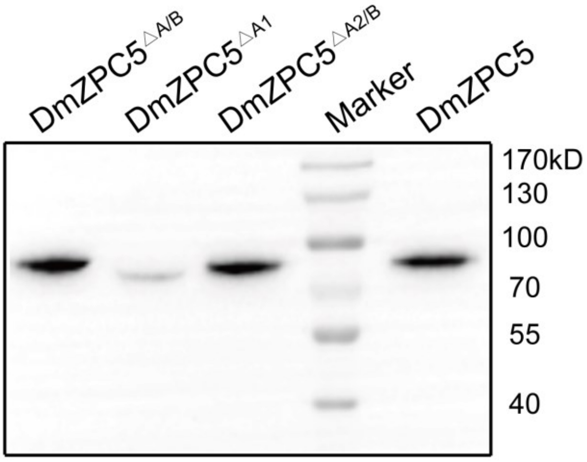

Supplementary Figure 7. The full-size Western blot picture supplement to Fig. 6e showing the sizes of the recombinantly expressed mutant and native DmZPC5s isolate from CHO cell lysates. The gel was run in SDS-PAGE under reducing conditions.

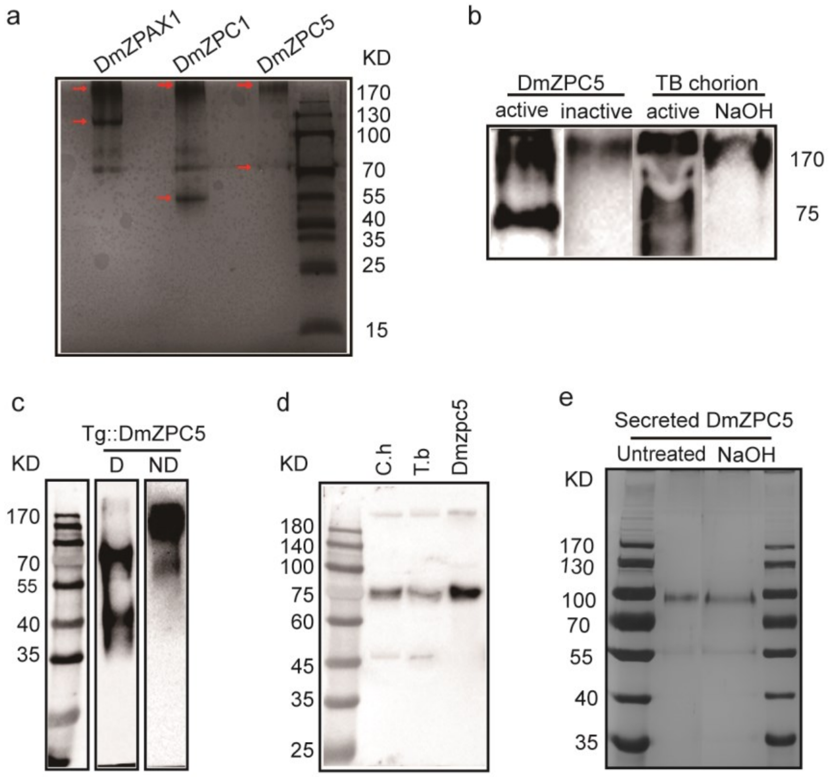

Supplementary Figure 8. A non-denaturing gel was used to analyze the polymerization status of the ice melting-promoting (IMP) activity in the AnnotoZP samples. The results indicate a correlation between IMP activity and the unpolymerized conformation of the protein. (a). Approximately 15 μg of the purified recombinant protein (DmZPAX1, DmZPC1, DmZPC5) was used. Each showed IMP activity, and each was loaded onto a non-denaturing PAGE gel and visualized using silver staining. By using an electrophoretic condition without adding denaturing agents to the gel or the loading buffer, the proteins were maintained in their native conformations. The presence of a band with the predicted molecular weight in addition to a larger band in each sample lane (indicated by red arrows) suggested the presence of monomers and polymers in each sample. **(b). Purified recombinant DmZPC5 and *T. bernacchii* (TB) chorion extracts with and without IMP activity demonstrated different conformational states.** The recombinant DmZPC5 sample containing IMP activity contained both polymers (>170 kD) and monomers (75 kD), while the inactive sample contained only the polymers. This was also true for the *T. bernacchii* chorion proteins, in which the active sample was found to contain two conformations, while the sample treated with NaOH that lost IMP activity showed only the polymers and not the monomers. **(c)**. The chorion proteins that were extracted from the DmZPC5 transgenic zebrafish

that has been found to have IMP activity possess non-cleaved (unpolymerized) DmZPC5 (the 75 kD band) under denaturing gel conditions (lane D), and both polymers and monomers when analyzed on a non-denaturing gel (lane ND). **(d). Western blot with anti-DmZPC5 antibody on a denaturing SDS-PAGE gel to show that the treatment of ZPC5 with 0.2M NaOH (final concentration) did not result in hydrolyzation of the ZP proteins. The denaturing gel electrophoresis and Western blotting were performed with the same protocol as other denaturing SDS-PAGE analysis. Lanes C. h and T. b is the total chorion proteins extracted from the eggs of** *C. hamatus* and *T. bernacchii*, while DmZPC5 is the purified recombinant protein. In all three cases, NaOH treated samples contained the intact full-length 75 kD DmZPC5. The 45 kD bands appeared in the C. h and T. b lanes are the ZP products naturally present in the egg chorion resulted from the cleavage of the precursor protein at the furin cleavage site evidenced by the presence of the same 45KD band in the same samples without NaOH treatment shown in Supplementary Fig. 2b. The ChZPC5, TbZPC5 are highly similar to DmZPC5 in protein sequence, and thus can be detected by the DmZPC5 antibody. **(e). Denaturing SDS-PAGE gel analysis of the secreted DmZPC5 after treating with 0.2M NaOH.** About 2 mg ml^{-1} of DmZPC5 purified from the transfected CHO culture medium was treated with 0.2M NaOH for 30 minutes (the ‘NaOH’ lane) and loaded on a denaturing SDS-PAGE, with the same amount of untreated secreted dmZPC5 (the ‘untreated’ lane) as control. Silver staining was used to visualize the protein. Again, no significant level of degraded protein was seen in the NaOH treated sample.

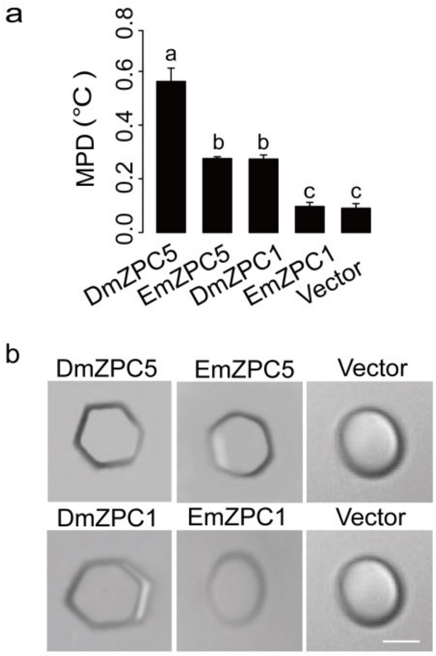

Supplementary Figure 9. (a). Enhanced melting point depression was observed in the recombinant ZPC1 and ZPC5 of the Antarctic notothenioid species *D. mawsoni* (DmZPC1, DmZPC5) compared to the MPDs of the orthologs of the basal temperate notothenioid species *E. maclovinus* (EmZPC1, EmZPC5). **(b).** Analysis of ice morphology revealed that a sharper hexagonal surface was associated with the DmZP protein samples. All of the proteins were assessed at a concentration of 3 mg ml^{-1}. Scale bar, 25 μm.

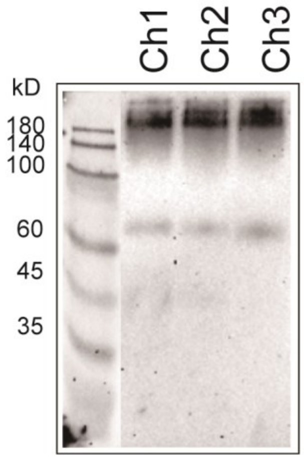

Supplementary Figure 10. **Western blotting analysis of serum total proteins of *C. hamatus* on**

a non-denaturing PAGE gel showing presence of ZPC5 monomers *in vivo*. The anti-DmZPC5 antibody was used for detection of homologous proteins. The concentration of total ZPC5 (including monomers and polymers) in the serum was estimated to be 2 mg ml^{-1}. Ch1, Ch2, and Ch3 represent three individuals of C. hamatus: Ch2 is a male, and the other two are female judged from their developed gonads.

Supplementary Table 1

GenBank accession number

Species / Gene	*D.mawsoni*	*N.coriiceps*	*C.hamatus*	*G.acuticeps*	*T.bernacchii*	*N.angustata*	*D.eleginoides*	*L.nudifrons*	*E.maclovinus*	*O.latipes*
ZPAX1	KM225616	KU517856	KU517856	KU499996<br>KU522422<br>KU522423	KU306106		KU500004	KU530185	KU306105	AF128807
ZPAX2	KU306107	KU499993	KU517857<br>KU517858<br>KU517859<br>KU517860	KU530176	KU306109			KU530184	KU306108	
ZPC1	KM030015	KU512752	KM076760<br>KU517863<br>KU517864<br>KU517865	KM076762<br>KU499997<br>KU530175	KM030017<br>KU306084	KU512751	KU512745	KU530178	KM030016<br>KU306083	AF128809
ZPC2	KM225617	KU500005	KU517866<br>KU517867	KU499999<br>KU530174	KU306086		KU500003	KU530179	KU306085	AF128810
ZPC3	KU306087	KU499995		KU500001<br>KU522425	KU306089<br>KU306090			KU530180	KU306088	AF128811
ZPC4	KU306091		KU517868<br>KU517869	KU499998<br>KU522426	KU306093			KU530181	KU306092	AF128812

ZPC5	KM030018<br>KU306094<br>KU306095<br>KU306096<br>KU306097<br>KU306098<br>KU306099<br>KU306100	KU512753<br>KU512754<br>KU512755<br>KU512756	KM076761<br>KU517870<br>KU517871<br>KU517872	KM076763<br>KU500002<br>KU522427<br>KU522428	KM030020<br>KU306103	KU500006	KU512746<br>KU512747<br>KU512748<br>KU512749<br>KU512750	KU530177	KM030019<br>KU306101<br>KU306102	AF128813
ZPB	KU306080	KU499994	KU517862	KU500000<br>KU522424	KU306082			KU530183	KU306081	AF128808
ZPD	KM225618		KU517873	KU522429	KU306104			KU530182		
CGL	KU306079									AB025967
CGH	KU306078									D89609

RNA seq data

SRP068525 transcriptomes data form *T. bernacchii* liver, brain and ovary tissues

GSE70113 transcriptome data from *G. acuticeps*, *T. bernacchii*, *C. hamatus* head kindey

Supplementary Table 2. Copy number of each ZP type per haploid genome deduced from real-time PCR.

Species	Gene copy numbers per haploid genome (mean±SD)							
	**ZPC1**	**ZPC2**	**ZPC3**	**ZPC5**	**ZPAX1**	**ZPAX2**	**ZPB**	**Total**
D.mawsoni	8.84±0.64	12.76±0.17	3.52±0.16	15.04±0.55	7.32±0.76	6.94±0.16	5.71±0.21	60.13
N.coriiceps	5.14±0.08	23.24±0.81	3.12±0.08	25.31±0.99	19.98±1.56	23.60±1.83	6.53±0.59	106.92
C.hamatus	6.05±0.06	2.63±0.02	12.17±0.15	4.96±0.66	20.55±0.51	5.36±0.11	8.26±0.36	59.98
G.acuticeps	3.92±0.05	7.28±0.04	10.62±0.04	17.64±0.06	37.11±0.53	Not detected	0.59±0.02	77.16
T.bernacchii	3.84±0.53	23.93±2.83	31.98±0.71	34.52±1.28	9.70±1.13	3.34±0.38	0.35±0.02	65.97
N.angustata	1.45±0.11	12.62±0.59	3.19±0.09	9.96±0.06	3.60±0.46	6.40±0.93	3.30±0.37	40.52
D.eleginoides	5.73±0.16	9.38±0.31	3.69±0.12	11.25±0.17	6.94±0.22	7.32±0.16	5.72±0.33	50.03
L. nudifrons	2.35±0.25	6.45±0.09	6.37±0.22	3.26±0.16	1.76±0.13	Not detected	Not detected	20.19
E.maclovinus	1.05±0.05	1.18±0.12	0.86±0.02	1.39±0.06	1.36±0.08	1.16±0.12	1.10±0.05	8.1
**B. variegatus*	0.84	0.36	1.57	0.96	1.24	0.82	0.97	6.76
***O.latipes*	1	1	1	1	1	1	1 (10)	6

*The ZP copy number of *B. Variegatus* was estimated from the gene ratios derived from the aCGH results in Chen Z. et al 2008

** The ZP copy number of *O.latipes* was derived from the reference of Kanamori A . et al 2003

The colors represent the different thermal environments of the fishes, same as in Fig. 2a

Supplementary Table 3. The geographic localities of the *Notothenioids* sampled in this study.

Species	Family	Locality (latitude, longitude)
Dissostichus mawsoni	Nototheniidae	McMurdo Sound, Ross Sea (77°51’S, 166°40’E)
Notothenia coriiceps	Nototheniidae	Palmer Archipelago (64°51’S, 63°34’W)
Chionodraco hamatus	Channichthyidae	Davis Station, Antarctica (68°35’S, 77°58’E)
Gymnodraco acuticeps	Bathydraconidae	Zhongshan Station, Antarctica (69°22’S, 76°22’E)
Trematomus bernacchii	Nototheniidae	Zhongshan Station , Antarctica(69°22’S, 76°22’E)
Notothenia angustata	Nototheniidae	Otago Harbor, New Zealand (45°30’S, 170°E)
Dissostichus eleginoides	Nototheniidae	Falkland Islands (51°25’S, 57°35’W)
Lepidonotothen nudifrons	Nototheniidae	Ushuaia Argentina (54°47’S, 68°20’W)
Eleginops maclovinus	Eleginopidae	Falkland Islands (51°25’S, 57°35’W)

Supplementary Table 4: Primers used in this study

Primer name	Primer sequence(5'-3')	Purpose
**Ⅰ. Primers for amplification of the ZP genes from the genomic DNA.**		
ZPC1-F	ATGATGGTTTCATTTTGTCAAGGTG	Amplify the full length gene of ZPC1
ZPC1-R	TCAGGAATTCACATCTGTGATGGT	
ZPAX1-ZPF	CCACCCCAATGGCACAATGA	Amplify the ZP domain region of ZPAX1 genes
ZPAX1-ZPR	ATGCACACACTGGCCTCTGC	
ZPC2-ZPF	AACTGTGAAAAAGACTCCATAAGC	Amplify the ZP domain region of ZPC2 genes
ZPC2-ZPR	CATAGTGGCAAGCTTTCTTG	
ZPC5-NF	ATGGAGGCCTTTAATTTTCA	Amplify the Exon1 of ZPC5 genes
ZPC5-NR	GACACTGGGTAACTGAGGAC	
ZPC5-CF	ATGCCTTCAGATTTTATAATGAGGACAGAG	Amplify the C-terminus of ZPC5 genes
ZPC5-CR	CCATTGTTTCAGAAAGGTCAGATACTGTT	
ZPC5-ZPF	GCAGGTGAATACCATCAGAG	Amplify the ZP domain region of ZPC5 genes
ZPC5-ZPR	AGGTCTGCTGATGGTAATGA	
ZPC3-F	TTGGAGCCACAGTCTTGTAG	Amplify portion of the ZP domain region of ZPC3 genes
ZPC3-R	GGAAGGGAATATAAAACTGC	
ZPC4-F	CGACAAAAGAGAAGATTATC	Amplify portion of the ZP domain region of ZPC4 genes
ZPC4-R	TGCTCGATAAAGTCATATCT	
ZPB-F	TTGGACCCAGAGGATCGATC	Amplify portion of the ZP domain region of ZPB genes
ZPB-R	ACCCATCAACCAGGAGGTCC	
ZPAX2-F	GAGTGTTTCCCTAATGGGACC	Amplify portion of the ZP domain region of ZPAX2 genes
ZPAX2-R	TACGGTTCACTCCTGCGAGG	
EmZPC3/SP	ACATTAAGATTTGACACAGAACGTC	Amplify portion of ZPC3 gene from *E. maclovivus* genomic
EmZPC3/ASP	CTGGTCGTGCCACAGGAGTT	DNA

TbZPAX1-SP	ATCCATCCTACGAGCTCTTC	Amplify portion of ZPAX1 gene from *T. bernacchii* genomic
TbZPAX1-ASP	CATAAAGGGTTGCCCAACAG	DNA
TBZPC2-SP	AAAAAATAACAGGGGTAGCG	Amplify portion of ZPC2 gene from *T. bernacchii* genomic
TBZPC2-ASP	CACATTGCTGTTAGCCTGCA	DNA
**Ⅱ.Primers for 3'-RACE and 5'-RACE of Em ZPC1 and ZPC5 genes.**		
EmZPC5-GSP1	ATCACATGGGGCTCCGAGTGTT	Amplify the 3' end of Em ZPC5 gene
EmZPC5-GSP2	GACTCTCAGCTTCCAGGCTCAA	
EmZPC1-GSP1	ATGCAGACATGAAAGTGGACTG	Amplify the 5' end of Em ZPC1 gene
EmZPC1-GSP2	AGACCCATCAGCCCTTGCTGCT	
EmZPC1-GSP3	ACTGCAAACTTTTGGCTTGGGA	
EmZPC1-GSPR1	ATCCCAAGCCAAAAGTTTGCAG	Amplify the 3' end of Em ZPC1 gene
EmZPC1-GSPR2	TCAAGAAGCAGCAGCAAGGGCT	
**Ⅲ. Primers for quantification the ZP gene copy numbers in different fish species by real time quantitative PCR.**		
ZPAX1-QF(cdn)	TCCTGCAGATCCATCCTACGA	Amplify the ZPAX1 gene in *D. mawsoni, D. eleginoides N.*
ZPAX1-QR(dn)	CAGAGGCTGCCGCAGATATT	*coriiceps, and N. angustata*
ChZPAX1-QR	AAAATACAGAGGCTGCTGCAGATA	Paired with ZPAX1-QF to amplify the ZPAX1 gene in *C.hamatus*
EmZPAX1-QF	CGGGACTCTTCTCCTGCAGAT	Amplify the ZPAX1 gene in *E. maclovivus*
EmZPAX1-QR	GGCTGCCGCAGATATTTCA	
ZPC1-QF(ce)	CAGGCCCCGCTGAATCTAC	Amplify the ZPC1 gene in *C.hamatus*
ZPC1-QR(c)	TCTGCACCACACTAGCCATGAT	
EmZPC1-QR	TCTGGTCCACACTAGCCATGAT	Paired with ZPC1-QF(ce) to amplify the ZPC1 gene in *E. maclovinus*
NcZPC1-QF	CAGGCCCTGCTGAATCTACAAG	Amplify the ZPC1 gene in *N. coriiceps* and *N. angustata*

NcZPC1-QR	TCTGCACCACACTAGCCATGA	
DmZPC1-QF	CAGGCCCTGCTGAATCTACAC	Amplify the ZPC1 gene in *D. mawsoni, D. eleginoides* and *T. bernacchii*
DmZPC1-QR	GCAAGGGCTGATGGGTCTT	
ZPC2-QR(de)	CTCTCCGCTGGGCAGAAC	Amplify the ZPC2 gene in *D. mawsoni* and *D. eleginoides*
DmZPC2-QF	CGGCAATATGCAGCTCGTTAC	
EmZPC2-QF	GCCATATGCAGCTCGTCTTTT	Paired with ZPC2-QF(de) to amplify the ZPC1 gene in *E. maclovinus*
ChZPC2-QF	CGATGCTGGTGCCATTTG	Amplify the ZPC2 gene in *C.hamatus*
ChZPC2-QR	CTCCGCTGGGCAGAACAT	
NcZPC2-QF	AGCAATATGCAGCTCGTCACTTC	Amplify the ZPC2 gene in *N. coriiceps* and *N. angustata*
NcZPC2-QR	CCTCTCCCCTGGGCAGAA	
ZPC5-QF(dc)	CCGCAGCAGGTGAATACCAT	Amplify the ZPC5 gene in *T. bernacchii, D. mawsoni* and *D. eleginoides*
DmZPC5-QR	GCAAACATGTCGGCTTTGATAAC	
ChZPC5-QR	GCGTATCTCGTCACCATCAACA	Paired with ZPC5-QF(dc) to amplify the ZPC5 gene in *C.hamatus*
EmZPC5-QF	ACGACCGCAAGTGAATACCAT	Amplify the ZPC5 gene in *E. maclovivus*
EmZPC5-QR	CGCAAACATGTCGGCTTTT	
NcZPC5-QF	TCATCCAGACTCTTTGGAGATTGTT	Amplify the ZPC5 gene in *N. coriiceps* and *N. angustata*
NcZPC5-QR	AGGCGTATCTCGTCACCATCA	
ChZPAX2-QF	GGTCCCACATACAGCAACGA	Paired with ZPAX2-QR to amplify the ZPAX2 gene in *C.hamatus*
ZPAX2-QF	GCCCCACATACAGCAACGA	Amplify the ZPAX2 gene in *N. coriiceps*, *N. angustata, D. mawsoni* and *D. eleginoides*
ZPAX2-QR	CGTCCCACAGGAGTTTGCA	
EmZPAX2-QF	TGGTCCGACATACAGCAATGA	Amplify the ZPAX2 gene in *E. maclovinus*
EmZPAX2-QR	CTGGTCGTGCCACAGGAGTT	
EmZPC3-QF	TGCACAGAAGCGTCCATGAT	Amplify the ZPC3 gene in *E. maclovinus*

EmZPC3-QR	AAAGGCGTCCATTGTTGAAGA	
EmZPC4-QF	TTTCTGCCATCATGGGTAACC	Amplify the ZPC4 gene in *E. maclovinus*
EmZPC4-QR	GCAACACAATGGTCCACATAGACT	
ZPB-QF	TGCTGAGGGAACCTGTCTATGTT	Amplify the ZPB gene in *N. coriiceps*, *N. angustata*, *D.*
ZPB-QR	CAGGACGATGTTTGGGTCAGA	*mawsoni, D. eleginoides, T. bernacchii* and *C.hamatus*
EmZPB-QF	CCAGCAGACTACCCAGTCACTAAA	Amplify the ZPB gene in *E. maclovivus*
EmZPB-QR	CCAGGATGCTCACCTCAACAT	
ZPC4-QF	TTTTCACGTTTCTGCCTTCATG	Amplify the ZPC4 gene in *N. coriiceps* and *N. angustata*, *D.*
ZPC4-QR	GCAACACAATGGTCCACATAGACT	*mawsoni* and *D. eleginoides*, *C.hamatus*, *T. bernacchii*
DmZPC3-QF	CCTTTTGTCTCCATGGGATTTTC	Amplify the ZPC3 gene in *D. mawsoni*
DmZPC3-QR	TCGACACTGACTGCTCTGTGAA	
GaZPC1-QF	TGCTGTACGGCAATGATCTGA	Amplify the ZPC1 gene in *G. acuticeps*
GaZPC1-QR	GGGTGAGTAAAGGCCAAATTGT	
GaZPC2-QF	CGCACTCGATGGGATACACA	Amplify the ZPC2 gene in *G. acuticeps*
GaZPC2-QR	GACTTTCAGGCCCAGACCAA	
GaZPC3-QF	ACACTGACTGCTCTGTGAATGCTT	Amplify the ZPC3 gene in *G. acuticeps*
GaZPC3-QR	GCCTTTTGTCTCCATGGGATT	
GaZPC4-QF	ATCCCCTCTCAAACTGCCAAT	Amplify the ZPC4 gene in *G. acuticeps*
GaZPC4-QR	ATGACTTGTTTACGTTTGGATTTCTCT	
GaZPC5-QF	TGTTAGCTCCTCTTTTGCTTTCCT	Amplify the ZPC5 gene in *G. acuticeps*
GaZPC5-QR	CTGTGTTCGCAAGGCTCTGA	
GaZPAX1-QF	CACCCCAATGGCACAATGA	Amplify the ZPAX1 gene in *G. acuticeps*
GaZPAX1-QR	TGGCTTCAGATTACGAGTCGATT	
GaZPB-QF	CCTCCATGACGATAGCTTCCA	Amplify the ZPB gene in *G. acuticeps*
GaZPB-QR	TCCGCACAGGTTATTGTTTCAG	
LnZPAX1-QR	GACCACATAGAAGCAGGAAA	Amplify the ZPAX1 gene in *L. nudifrons*

LnZPAX1-QR	AAAGGAGCAGCCAACACAT	
LnZPC1-QF	GTTTCTCACGTTGTTTACCC	Amplify the ZPC1 gene in *L. nudifrons*
LnZPC1-QR	CATTTTCTACACATTTTGCTT	
LnZPC2-QF	GCATGAGCCCTCACCATTT	Amplify the ZPC2 gene in *L. nudifrons*
LN-ZPC2-QR	TCCTCCTTTCCTGAACCCG	
LnZPC3-QF	GAGTCACCAGCAGCAACAG	Amplify the ZPC3 gene in *L. nudifrons*
LnZPC3-QR	TCAGCGTACAATGCACAGA	
LnZPC5-QF	GTGTTGTTCGAATGGAGGAGGCT	Amplify the ZPC5 gene in *L. nudifrons*
LnZPC5-QR	GTTGACATGAAGGGGATCCAGGTAG	
TbZPC2-QF	CAATATGGGCAACAGTGGAG	Amplify the ZPC2 gene in *T. bernacchii*
TbZPC2-QR	ACACATTCCTCCATGAGCAG	
TbZPAX1-QF	GGTGGAGCTGATGCATTCTA	Amplify the ZPAX1 gene in *T. bernacchii*
TbZPAX1-QR	AGGGTTGCCCAACAGTTCT	
TbZPC3-QF	TCACAGAGCAGTCAGTGTCG	Amplify the ZPC3 gene in *T. bernacchii*
TbZPC3-QF	GAGCCACAGTCTTGTAGTCCAG	

Ⅳ.Primers for construction of the transgenic plasmids

Tol2-5'F	CCATTAATCAGAGGTGTAAAGTACTTGAGTAATT	Amplify the 5' end of Tol2 gene
Tol2-5'R	CACGCTAGCTAATAGCAAGGGAAAATAGAATGAAGT	
Tol2-3' F	GACTTAAGCAGAGGTGTAAAAAGTACTCAAAAATTTTACTCAAGTGAAA	Amplify the 3' end of Tol2 gene
Tol2-3' R	GACTTAAGGCTTAAACAAGAATCTCTAGTTTTC	
ZB-ZP3-pro F	GCACTCGAGTGGTATTATAGGAAGTAAAACCA	Amplify the Zebrafish ZP3 promoter
ZB-ZP3-pro R	ACGAATTCATTGCCTGCTGACTAATTAAACC	
DmZPC5-F	CTTCGAATTCTTTCAGTTTCACTTATGGAG	Construction of DmZPC5 transgenic vector
DmZPC5-R	TGGATCCCCGGGAATAATTTATTTTCTCTTTGGGTC	

**Ⅴ.Primers for quantification of D.mawsoni mRNA by real time quantitative PCR.**		
ZPAX1-QF(cdn)	TCCTGCAGATCCATCCTACGA	Amplify the ZPAX1 gene in *D. mawsoni*
ZPAX1-QR(dn)	CAGAGGCTGCCGCAGATATT	
ZPB-QF	TGCTGAGGGAACCTGTCTATGTT	Amplify the ZPB gene in *D. mawsoni*
ZPB-QR	CAGGACGATGTTTGGGTCAGA	
ZPAX2-QF	GCCCCACATACAGCAACGA	Amplify the ZPAX2 gene in *D. mawsoni*
ZPAX2-QR	CGTCCCACAGGAGTTTGCA	
ZPC5-QF(dc)	CCGCAGCAGGTGAATACCAT	Amplify the ZPC5 gene in *D. mawsoni*
Dm-ZPC5-QR	GCAAACATGTCGGCTTTGATAAC	
ZPC2-QR(de)	CTCTCCGCTGGGCAGAAC	Amplify the ZPC2 gene in *D. mawsoni*
DmZPC2-QF	CGGCAATATGCAGCTCGTTAC	
ZPC4-QF	TTTTCACGTTTCTGCCTTCATG	Amplify the ZPC4 gene in *D. mawsoni*
ZPC4-QR	GCAACACAATGGTCCACATAGACT	
Bactin-QF	ATTGTGACCAACTGGGATGA	Amplify the beta-actin gene in *D. mawsoni*
Bactin-QR	GGGCAACTCTCAGCTCGT	
**Ⅵ.Primers for construction of the expression plasmids**		
EmZPC1/SP	TCAGATCTCGAGATTCAGTCAAAATGGCTTCC	Construction of EmZPC1 expression vector
EmZPC1/ASP	CCCGGGCCGCGGAACCAAGAATAAAATCCCAATTCA	
DmZPC1/SP	TCAGATCTCGAGGTCATGATGGTTTCATTTTG	Construction of DmZPC1 expression vector
DmZPC1/ASP	CCCGGGCCGCGGGAATAAAAACCAAATTCAGATTGA	
DmZPC2-F	CTTCGAATTCATGGGGACTCAACTCTACCT	Construction of DmZPC2 expression vector
DmZPC2-R	ACGACGTCGACCAACTTTAATCTGCATATGG	
DmZPC5-F	CTTCGAATTCTTTCAGTTTCACTTATGGAG	Construction of DmZPC5 expression vector
DmZPC5-R	TGGATCCCCGGGAATAATTTATTTTCTCTTTGGGTC	
EmZPC5-SP	CTTCGGAATTCATGGAGGCCTTTAATTTTCAGG	Construction of EmZPC5 expression vector

EmZPC5-ASP	CCCGGGTTATCTCTTTGGGTCCTTTG	
DmZPAX1-F	GACGAATTCGCTGCTAACAAACTGCAACATGCTGAG	Construction of DmZPAX1 expression vector
DmZPAX1-R	GACACTCCGCGGGTGCCAAACAAAACAATGAACAAA	
DmZPB-F	GACGTCGACGACTTATACCATAATGGAGC	Construction of DmZPB expression vector
DmZPB-R	GACACTCCGCGGTTCCAGTGGCTTTTCATCTC	
Q5SDM_DmZPC1_F	gatgatgataaaGACATGAAAGTGGACTGTGG	Insert FLAG into the DmZPC1 expression vector
Q5SDM_DmZPC1_R	atctttataatcTGCATATACCGCCATGGC	
Q5SDM_EmZPC1_F	gatgatgataaaTGAAACTGGACTGTGGGC	Insert FLAG into the EmZPC1 expression vector
Q5SDM_EmZPC1_R	atctttataatcTCTGCAAATACCGCAATG	
Q5SDM_dmZPAX1_F	gatgatgataaaAGGTCTAATGTGAAGCTAAATTTG	Insert FLAG into the DmZPAX1 expression vector
Q5SDM_dmZPAX1_R	atctttataatcGGCTTGACCCAAGATGATAAC	
Q5SDM_dmZPC2_F	gatgatgataaaGAAATCAAAGTAAACTGTGAAAAAGAC	Insert FLAG into the DmZPC2 expression vector
Q5SDM_dmZPC2_R	atctttataatcTGCATTAGCAACCGTTGTTG	
Q5SDM_dmZPB_F	gatgatgataaaCAGAATCCCTGGATGCAAC	Insert FLAG into the DmZPB expression vector
Q5SDM_dmZPB_R	atctttataatcGGCAAACGCATCACAGGC	
Q5SDM_EmZPC5_F	gatgatgataaaTTCCCGCCCACACGGTAC	Insert FLAG into the EmZPC5 expression vector
Q5SDM_EmZPC5_R	atctttataatcAGCGAAAGAGGAGCTAACACAG	
Q5SDM_dmZPC5_F	gatgatgataaaTTCCCGCCCACACGCTAC	Insert FLAG into the DmZPC5 expression vector
Q5SDM_dmZPC5_R	atctttataatcAGCAAAAGAGGAGCTAACACAGAG	

Supplementary References

1. Chen Z*, et al.* Transcriptomic and genomic evolution under constant cold in Antarctic notothenioid fish. *Proceedings of the National Academy of Sciences of the United States of America* **105**, 12944-12949 (2008).
2. Kanamori A, Naruse K, Mitani H, Shima A, Hori H. Genomic organization of ZP domain containing egg envelope genes in medaka (Oryzias latipes). *Gene* **305**, 35-45 (2003).
